# Flavin Reductase Contributes to Pneumococcal Virulence by Protecting from Oxidative Stress and Mediating Adhesion and Elicits Protection Against Pneumococcal Challenge

**DOI:** 10.1038/s41598-017-18645-8

**Published:** 2018-01-10

**Authors:** Giora I. Morozov, Nurith Porat, Tatyana Kushnir, Hastyar Najmuldeen, Asad Adawi, Vered Chalifa-Caspi, Rachel Benisty, Ariel Ohayon, Ofir Liron, Shalhevet Azriel, Itai Malka, Shahar Dotan, Maxim Portnoi, Andrew A. Piotrowski, Daniel Kafka, Barak Hajaj, Tali Fishilevich, Marilou Shagan, Michael Tal, Ron Ellis, Donald A. Morrison, Andrea M. Mitchell, Timothy J. Mitchell, Ron Dagan, Hasan Yesilkaya, Yaffa Mizrachi Nebenzahl

**Affiliations:** 1 The Shraga Segal Department of Microbiology and Immunology and Genetics, Faculty of Health Sciences, Ben-Gurion University of the Negev, Beer-Sheva, Israel; 2 Pediatric Infectious Disease Unit, Soroka University Medical Center, Beer Sheva, Israel; 3 Department of Infection, Immunity & Inflammation, University of Leicester, Leicester, United Kingdom; 4 Department of Biology, College of Science, University of Sulaimani, Sulaymaniyah, Iraq; 5 National Institute for Biotechnology in the Negev, Ben-Gurion University of the Negev, Beer-Sheva, Israel; 6 NasVax, Ness Ziona, Israel; 7 Department of Biological Sciences, University of Illinois at Chicago, Chicago, IL USA; 8 Institute of Microbiology and Infection, College of Medical and Dental Sciences, University of Birmingham, Birmingham, United Kingdom; 9 Faculty of Health Sciences, Ben-Gurion University of the Negev, Beer-Sheva, Israel

## Abstract

Pneumococcal flavin reductase (FlaR) is known to be cell-wall associated and possess age dependent antigenicity in children. This study aimed at characterizing FlaR and elucidating its involvement in pneumococcal physiology and virulence. Bioinformatic analysis of FlaR sequence identified three-conserved cysteine residues, suggesting a transition metal-binding capacity. Recombinant FlaR (rFlaR) bound Fe^2+^ and exhibited FAD-dependent NADP-reductase activity, which increased in the presence of cysteine or excess Fe^2+^ and inhibited by divalent-chelating agents. *fla*R mutant was highly susceptible to H_2_O_2_ compared to its wild type (WT) and complemented strains, suggesting a role for FlaR in pneumococcal oxidative stress resistance. Additionally, *fla*R mutant demonstrated significantly decreased mice mortality following intraperitoneal infection. Interestingly, lack of FlaR did not affect the extent of phagocytosis by primary mouse peritoneal macrophages but reduced adhesion to A549 cells compared to the WT and complemented strains. Noteworthy are the findings that immunization with rFlaR elicited protection in mice against intraperitoneal lethal challenge and anti-FlaR antisera neutralized bacterial virulence. Taken together, FlaR’s roles in pneumococcal physiology and virulence, combined with its lack of significant homology to human proteins, point towards rFlaR as a vaccine candidate.

## Introduction

*Streptococcus pneumoniae* is an anaerobic aero-tolerant organism whose metabolism is sensitive to the presence of oxygen^1^. Both oxygen and its ultimate metabolic by-product, hydrogen peroxide^2^, react slowly with cellular macromolecules and do not exert prominent destructive effects^3^. However, interaction of H_2_O_2_ with ferrous ions (Fe^2+^) produces hydroxyl radicals by the Fenton reaction and these highly reactive oxygen species (ROS) are capable of destroying DNA, membrane lipids and proteins^4–8^. In contrast to Fe^2+^, the ferric ion (Fe^3+^), which is the stable form of iron since it is chemically inert at neutral pH, is considered non-toxic^9,10^. However, the reducing conditions in the cytoplasm can reduce Fe^3+^ to the toxic Fe^2+^ state. Among the reducing agents capable of reducing ferric iron are intracellular cysteine^11^, reduced FAD^2^ and to a lesser extent, glutathione, thioredoxin, NADH and NADPH^6^. In culture, pneumococci produce millimolar concentrations of H_2_O_2_, as part of their normal metabolism^12^. Accordingly, *S*. *pneumoniae* is relatively resistant to the destructive effects of H_2_O_2_ and ROS in comparison to other bacteria^13,14^ even though it lacks the major H_2_O_2_-degrading enzyme, catalase^15–17^. Two membrane associated, extracellular- and intracellular- thioredoxin pathways have been described, which prevent damages caused by ROS molecules^18,19^.

Since Fe^2+^ may exert toxic effects through the Fenton reaction, the level of Fe^2+^ in all organisms is tightly controlled by its binding to specific ligands^2,20,21^. Iron concentration in aqueous solution is extremely limited (10^−8^–10^−9^ M) and well below the range that supports microbial growth (~10^−6^ M)^22^. Iron concentration within the host is further restricted (up to 10^−24^ M) in order to suppress the generation of toxic ROS ensuring bacteriostasis at potential sites of infection^23^. As Fe^2+^ is essential for many biological processes, scarcity of iron within the host has initiated evolutionary war between host and pathogen, where successful pathogens express various mechanisms for iron acquisition^24^. The withholding of metals, such as iron, to effectively starve pathogens of essential elements is referred to as “nutritional immunity” and is an important facet of the innate immune system^25^.

Intracellular Fe^2+^ in the bound state is unable to interact with H_2_O_2_ and consequently ROS production is prevented. In eukaryotic organisms, Fe^2+^ is bound to intracellular heme-containing proteins, including ferritin, iron–sulfur proteins and extracellular iron-binding proteins, such as transferrin and lactoferrin^26^. In prokaryotes, bacterioferritin, ferritin and Dps have similar functions^27–29^. *S*. *pneumoniae* have been found to acquire free inorganic iron by the ABC transporter lipoproteins PiaA and PiuA^30^, Pit and SPD_1609^31,32^. In addition *S*. *pneumoniae* binds host Fe^2+^ transporter molecules such as transferrin, lactoferrin^33^ and haemoglobin^34^ to remove and utilize the Fe^2+^ associated with these proteins. However, the full extent of pneumococcal iron acquisition systems and their role in pneumococcal survival and virulence is not known in detail.

In our ongoing studies, we have demonstrated an age-dependent enhancement of antibody-response to a group of *S*. *pneumoniae* surface protein antigens^35^. One of these proteins, which we have previously named pneumococcal surface immunogenic protein B (PsipB)^35^, was recently annotated in TIGR4 as flavin reductase (FlaR) (WP_000580659.1). In this study, we attributed functions to FlaR in Fe^2+^ binding and NADP reductase activity and demonstrated its importance to pneumococcal resistance to H_2_O_2_. Moreover, we found that FlaR contributes to pneumococcal virulence not only in oxygen rich environments, but also in oxygen limited environments. In line with these results, we ascribe an additional function for surface-expressed FlaR as an adhesin. Finally, FlaR elicited protective immune response in mice against *S*. *pneumoniae*, implying it can serve as a candidate vaccine.

## Results

### FlaR bioinformatic analyses

To examine whether flavin reductase is ubiquitous among *S*. *pneumoniae* strains, the flavin reductase DNA sequence of the TIGR4 strain was compared to 29 completely sequenced genomes of *S*. *pneumoniae*. All 29 genomes were found to contain a highly similar locus to SP_RS 02775 [BLAST e-value < 10^−76^, >97% identity, >70% query coverage (>98% coverage in 27 of the genomes)].

A BLAST search carried out with WP_000580663.1 against all *S*. *pneumoniae* RefSeq proteins annotated as “flavin reductase” retrieved 63 proteins, which were subjected to multiple sequence alignment and a phylogenetic tree was constructed. The tree was used to select a representative set of 9 divergent proteins, one from each clade of the tree (Supplementary Fig. S1). The position of a conserved flavin reductase domain is indicated, as well as three positions of conserved cysteines (equivalent to positions 66, 72 and 84 of WP_000580663.1). It is worth noting that cysteine was present in the three conserved positions in all 63 proteins, except 3 cases of substitution to tyrosine in the first or second position (data not shown).

A BLASTP search of WP_000580663.1 vs. RefSeq proteins excluding *S*. *pneumonia*e showed highly similar hits from other Streptococci species (e value < 10^−22^, 36–87% identify, query coverage >79%). Significant hits were also received from a wide range of non-Streptococci species (minimal e value was 10^−28^), however none had a higher a percent identity than 39%. Using an e value cutoff of 0.001, no eukaryotic proteins were retrieved.

### Recombinant FlaR protein production

**Figure 1:**
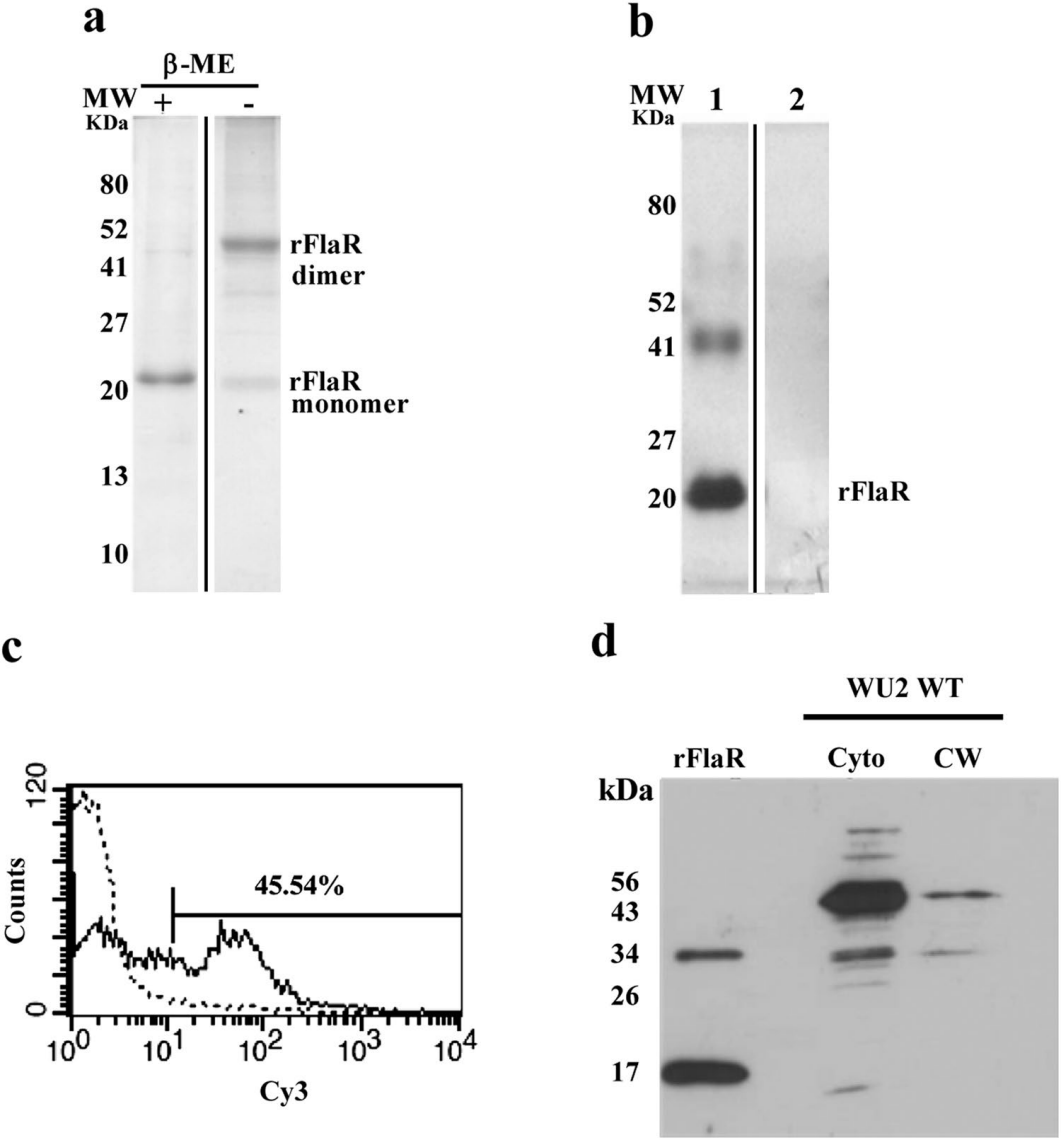
Expression of recombinant Flavin reductase (rFlaR). (**a**) rFlaR was purified using Ni-NTA beads under denaturing conditions and resolved by SDS-PAGE using sample buffer with or without β-mercaptoethanol (β-ME). (**b**) rFlaR was separated using SDS-PAGE. The protein was transferred to a nitrocellulose membrane and probed with rabbit antiserum raised against rFlaR (lane 1) or preimmune rabbit serum (lane 2). (**c**) Expression of FlaR on the surface of R6 was detected by flow cytometry using mouse anti-rFlaR antibodies. (**d**) Cytoplasmic (cyto) and cell wall (CW) fractions prepared from WU2 were subjected to electrophoresis and immunoblotted with rabbit anti-rFlaR antiserum. Of note, this gel was cropped from Supplementary Fig. S2.

rFlaR was overexpressed as HAT-tagged in *Escherichia coli* and purified under denaturing conditions. The theoretical molecular weight of the untagged protein was found to be 17.2 kDa. Separation of the HAT-tagged protein on reducing SDS-PAGE revealed a major band of 22 kDa and a minor band of 45 kDa (Fig. 1a). Non-reducing SDS-PAGE revealed the 45 kDa band to be the major band and 22 kDa to be the minor one (Fig. 1a). To identify the upper and lower bands were subjected to MALDI-TOF. Fourteen peptides, 9 derived from in-gel digestion of the lower band and 5 from the upper band, matched the database sequence of a hypothetical protein from *S*. *pneumoniae* R6 strain that is homologous to FlaR in the TIGR4 strain. The apparent molecular weight of the upper band was approximately double than that of the lower one, indicating that the upper band corresponds to a dimer of the lower band. Moreover, polyclonal antibodies, raised in rabbits against rFlaR, detected both bands by immunoblotting (Fig. 1b), none of which were detected by pre-immune sera.

### FlaR cellular localization

To validate the previous finding regarding the presence of FlaR on the bacterial surface^35^, we performed flow cytometry analysis on live R6 strain probed with a polyclonal mouse anti-rFlaR antibody. Indeed FlaR was found to be surface expressed (Fig. 1c). In addition, polyclonal rabbit antiserum recognized FlaR in the cytoplasm of WU2 WT strain in two forms: at ~17 kDa (untagged rFlaR monomer) and 34 kDa (untagged dimer; Fig. 1d). The 34 kDa band could also be observed in the bacterial cell-wall (CW) fraction (Fig. 1d) in accordance with our previous study^35^. A third, nonspecific band (~ 50kDa) was detected in the immunoblots of both the WU2 WT and WU2Δ*flaR* ^kan^ null mutant strains (Supplementary Fig. S2). The cytoplasmic protein, malonyl-CoA:ACP transacylase (FabD), which is involved in lipid metabolism^36^, was used as control for successful separation between CW and cytoplasmic fractions. FabD could be visualized (33 kDa) in both the total bacterial protein extract and the cytoplasm, but not in the CW fraction. This was lately described in Mizrachi Nebenzahl *et al.* 2016^37^.

### Refolding and Solubilization of rFlaR

**Figure 2:**
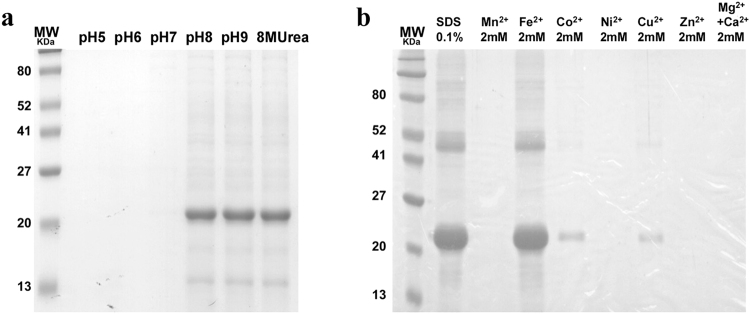
Refolding of rFlaR. (**a**) rFlaR was eluted from a Ni-NTA column under denaturing conditions and dialyzed against a buffer containing 0.1% SDS. The solution was then dialyzed against: lane 1, 20 mM acetate pH 5.0; lane 2, 20 mM MES pH 6.0; lane 3, 20 mM phosphate pH 7.0; lane 4, 20 mM phosphate pH 8.0; lane 5, 20 mM Tris pH 9.0. All buffers contained 150 mM NaCl. Lane 6, rFlaR before dialysis. The dialyzed solution was resolved on 1D SDS-PAGE and stained with Coomassie Brilliant Blue. (**b**) rFlaR, solubilized with buffer containing 0.1% SDS, was dialyzed against PBS containing 2 mM of the following metal salts: lane 1, rFlaR before dialysis; lane 2, 2mM MnCl_2_, lane 3, 2mM  FeSO_4_, lane 4, 2mM CoCl_2_, lane 5, 2mM NiCl_2_, lane 6, 2mM CuSO_4_, lane 7, 2mM ZnCl_2_, lane 8, 2mM MgCl_2_ and 2mM CaCl_2_. The dialyzed solution was resolved on 1D SDS-PAGE and stained with Coomassie Brilliant Blue.

The rFlaR was insoluble under physiological conditions and its purification could be obtained under denaturing conditions in buffer supplemented with 8 M urea. Different refolding conditions were tested for their ability to renature the protein. Dialysis of the denatured protein against buffers at different pH levels revealed that rFlaR could be solubilized at pH ≥ 8.0 (Fig. 2a). The presence of cysteine-rich sequence suggests that the protein has a transitional metal-binding ability^38^. Thus, rFlaR was dialyzed against PBS (pH 7.3) supplemented with 2 mM salts of either transition metal or calcium and magnesium (2 mM of each). rFlaR became fully soluble in the presence of 2 mM FeSO_4_. Partial solubility could be obtained in the presence of 2 mM CoCl_2_ or CuSO_4_ (Fig. 2b).

### FlaR is a Fe^2+^-binding protein

**Figure 3:**
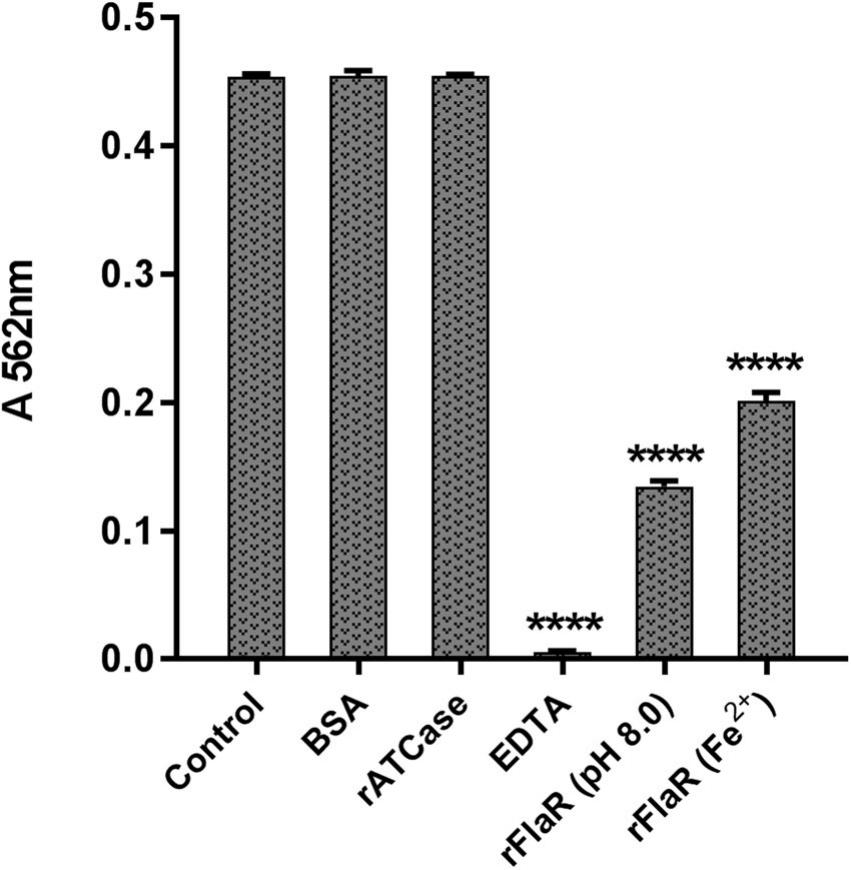
Iron binding by FlaR. The extent of Fe^2+^ staining by ferrozine was monitored in iron ammonium sulfate solution in PBS (control), PBS supplemented with BSA (BSA), PBS supplemented with rATCase (rATCase), EDTA, rFlaR refolded at pH 8.0 (rFlaR (pH 8.0)) and rFlaR refolded in the presence Fe^2+^ iron ions (Fe^2+^). The results presented are the mean of three different experiments (one way ANOVA with Dunnett’s post hoc multiple comparisons test, n = 3 in each group, ****p = 0.0001).

To further verify that rFlaR can directly bind Fe^2+^, solubilized rFlaR was incubated with ferrozine in the presence of free Fe^2+^ ions. Ferrozine is well known as an effective chelator of Fe^2+^ forming a complex that peaks at 562 nm^39^. During the experiment the absorbance was significantly lower in solutions containing rFlaR, solubilized either at pH 8 or with 2 mM Fe^2+^, in comparison to three different controls supplemented with 2 mM Fe^2+^: 1) PBS (Fig. 3; Control; p = 0.0001); 2) PBS supplemented with bovine serum albumin (BSA) and 3) PBS supplemented with pneumococcal protein aspartate carbomoyltransferase (WP_001293838.1; ATCase)^37^, not known to bind Fe^2+^. As a positive control for Fe^2+^ binding, we have used ethylenediaminetetraacetic acid (EDTA; 10 nM), which significantly inhibited the ferrozine-iron interaction (Fig. 3, p = 0.0001). The ability of rFlaR to compete with ferrozine for Fe^2+^ binding points out that FlaR is a Fe^2+^ binding protein.

### rFlaR has NADP reductase activity

**Figure 4:**
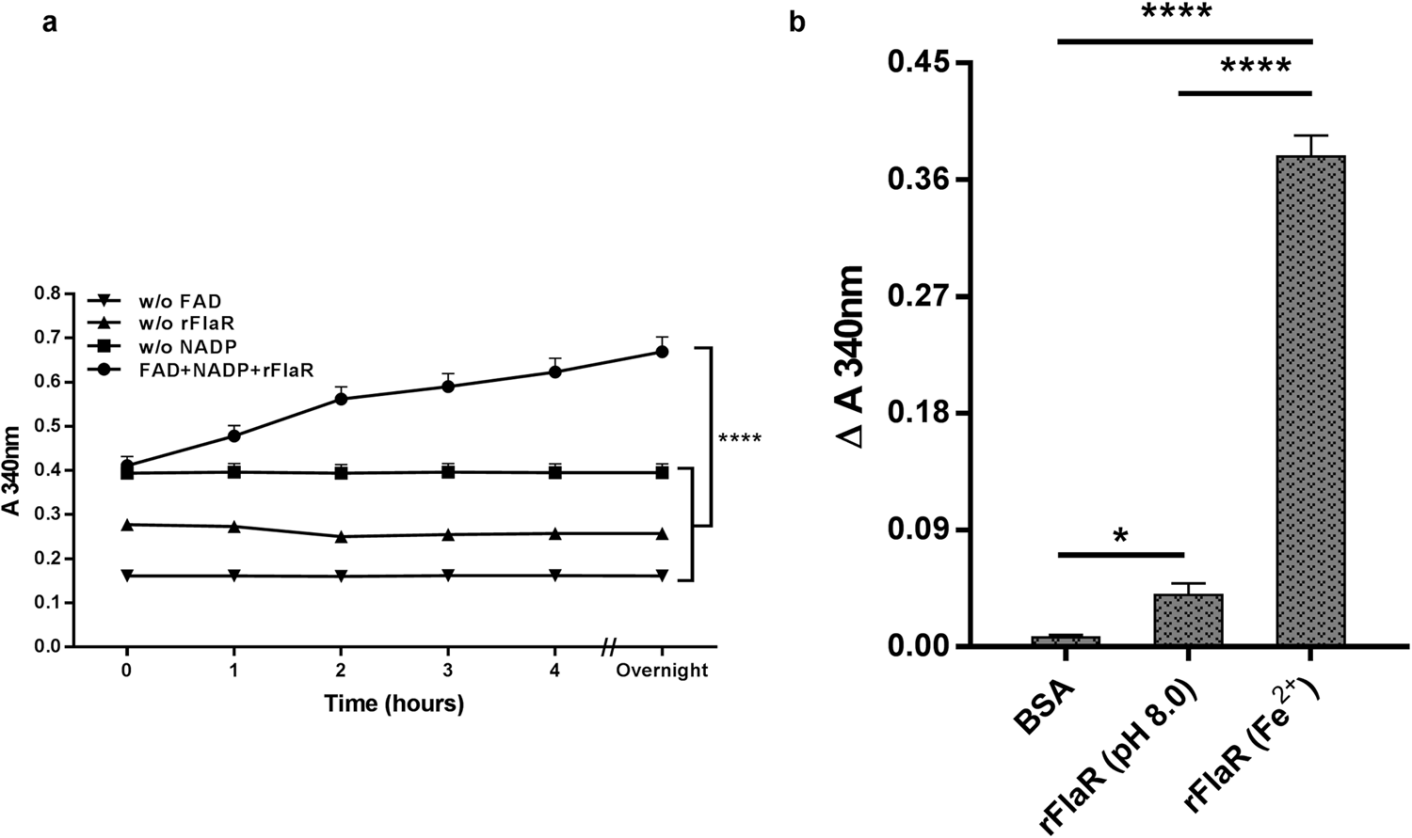
NADP-reductase activity of rFlaR. (**a**) A significant reduction of NADP by rFlaR refolded in the presence Fe^2+^ was measured over a period of 16 hours at A_340_ at the presence of FAD (Spearman correlation, r = 1, **p = 0.0014). The reaction mixtures without individual components (w/o rFlaR, w/o FAD, w/o NADP) served as controls and no redox activity could be detected (whole graph comparisons using one way ANOVA with Dunnett’s post hoc test, ****p = 0.0001). (**b**) NADP-reductase activities of rFlaR refolded at the presence (rFlaR (Fe^2+^)) and rFlaR refolded at pH 8.0 (rFlaR (pH 8)) at the absence of Fe^2+^ were compared. In the control reaction mixture rFlaR was replaced with BSA. The difference between the absorbance at 340 nm after 90 min incubation and the absorbance at 340 nm at time 0 (Δ A_340_ nm) was calculated. The results presented are the mean of three different experiments (one way ANOVA with Dunnett’s Post Hoc multiple comparisons test, n = 3 in each group, *p = 0.02; ****p = 0.0001).

Flavin-binding domains are found in proteins with oxidoreductase activity. Nicotinamide adenine dinucleotide (phosphate) (NAD(P)H) is used as an electron donor in the enzymatic reactions of flavin reductases^38–40^. To test whether rFlaR is involved in flavin-dependent redox reactions, we performed a flavin reductase activity assay as previously described^40,41^. Oxidation of NADPH, measured at A_340_ nm, could not be observed in the presence of iron-refolded rFlaR and flavin adenine dinucleotide (FAD) (data not shown). Significant increase in the reduction of NADP mediated by rFlaR was observed only in the presence of all the reaction components (NADP and FAD) over time (Fig. 4a, Spearman correlation, r = 1, p = 0.0014). No significant NADP reduction could be observed in the absence of either one of the components (Fig. 4a, p = 0.0001). After the first hour of the reaction in the presence Fe^2+^ and rFlaR, the absorbance change at 340 nm was 0.09, equivalent to 14.5 µM/hour of generated NADPH (calculated by using ε 6.22 mM^−1^ cm^−1^)^42,43^. Hence, rFlaR specific activity is 40.3 µM min^−1^ mg^−1^ protein. It should be noted that iron-refolded rFlaR exhibited significantly higher NADP-reductase activity compared to rFlaR refolded at pH 8.0 in the absence of Fe^2+^ ions. BSA was used as a negative control (Fig. 4b, p = 0.0001). The NADP reductase activity of rFlaR refolded at pH 8.0 was still significantly higher in comparison to the negative control (Fig. 4b, p = 0.02).

### rFlaR NADP reductase activity is Fe^2+^ dependent

**Figure 5:**
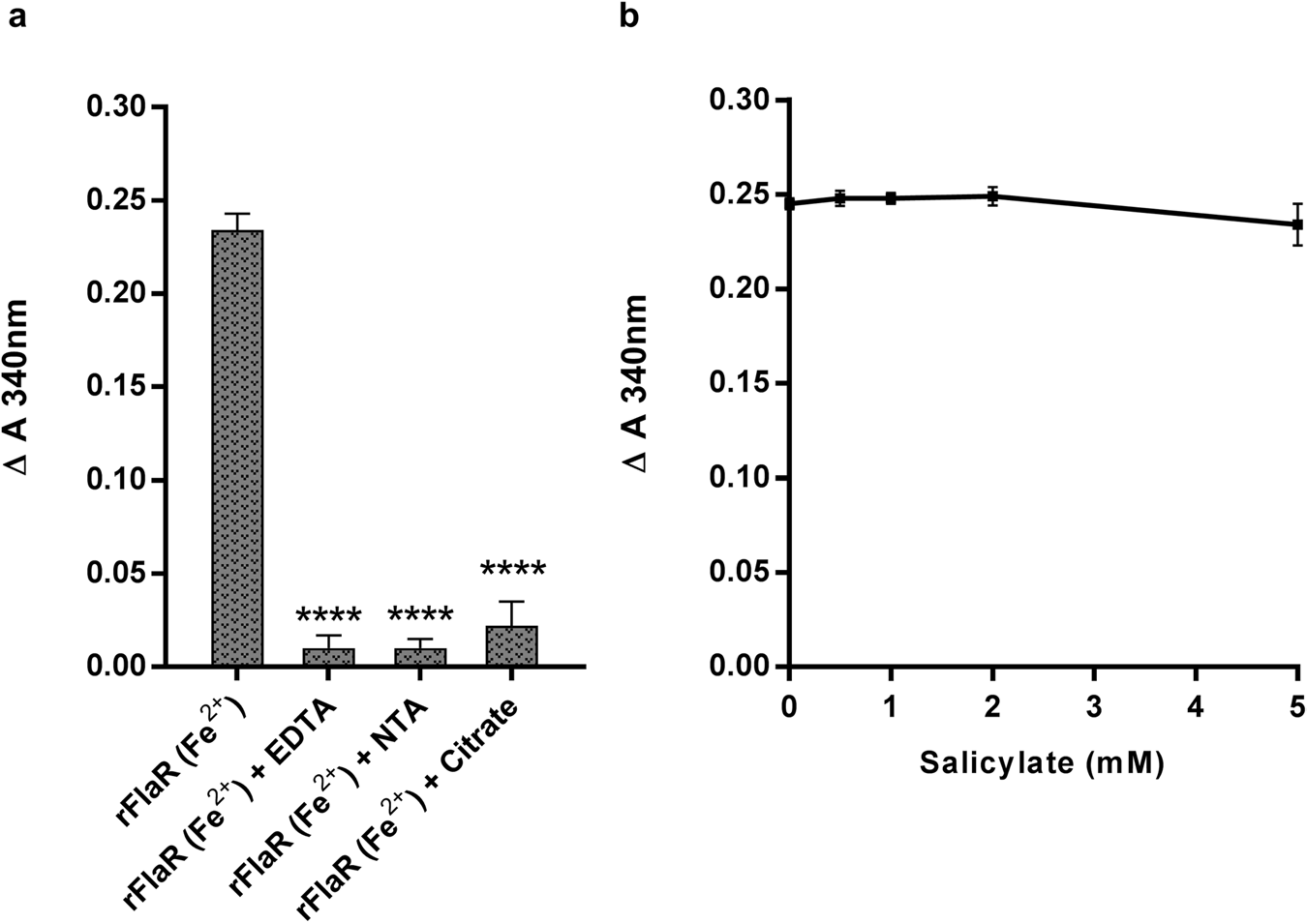
Fe^2+^ involvement in FlaR enzymatic activity. (**a**) Different divalent ion chelators (EDTA (10 nM), NTA (10 nM) and citrate (10 nM)) introduced into rFlaR reaction mixture significantly inhibited rFlaR redox enzymatic activity. Results are presented as Δ A_340_ nm, that was calculated as described in Fig. 3 The results presented are the mean of three different experiments (one way ANOVA with Dunnett’s post hoc multiple comparisons test, n = 3 in each group, ****p = 0.0001). (**b**) The effect of the H_2_O_2_ on rFlaR NADP-reductase activity was eliminated by H_2_O_2_ scavenger, salicylate (Spearman correlation, r = −0.0513, p = 0.5 not significant).

To evaluate the importance of Fe^2+^ for rFlaR enzymatic activity, different divalent ion chelators were introduced into the reaction mixture. EDTA (10 nM), Nitrilotriacetic acid (NTA; 10 nM) and citrate (10 nM) all significantly inhibited rFlaR NADP reductase activity (Fig. 5a; p = 0.0001). To rule out the possibility that Fe^2+^ is oxidized by H_2_O_2_, NADP reductase activity was measured in the presence of the H_2_O_2_ scavenger, salicylate. Indeed, salicylate did not inhibit NADPH production by rFlaR (Fig. 5b), indicating that H_2_O_2_ does not affect FlaR activity directly.

### Fe^2+^ is the electron donor in the rFlaR-NADP reductase activity

**Figure 6:**
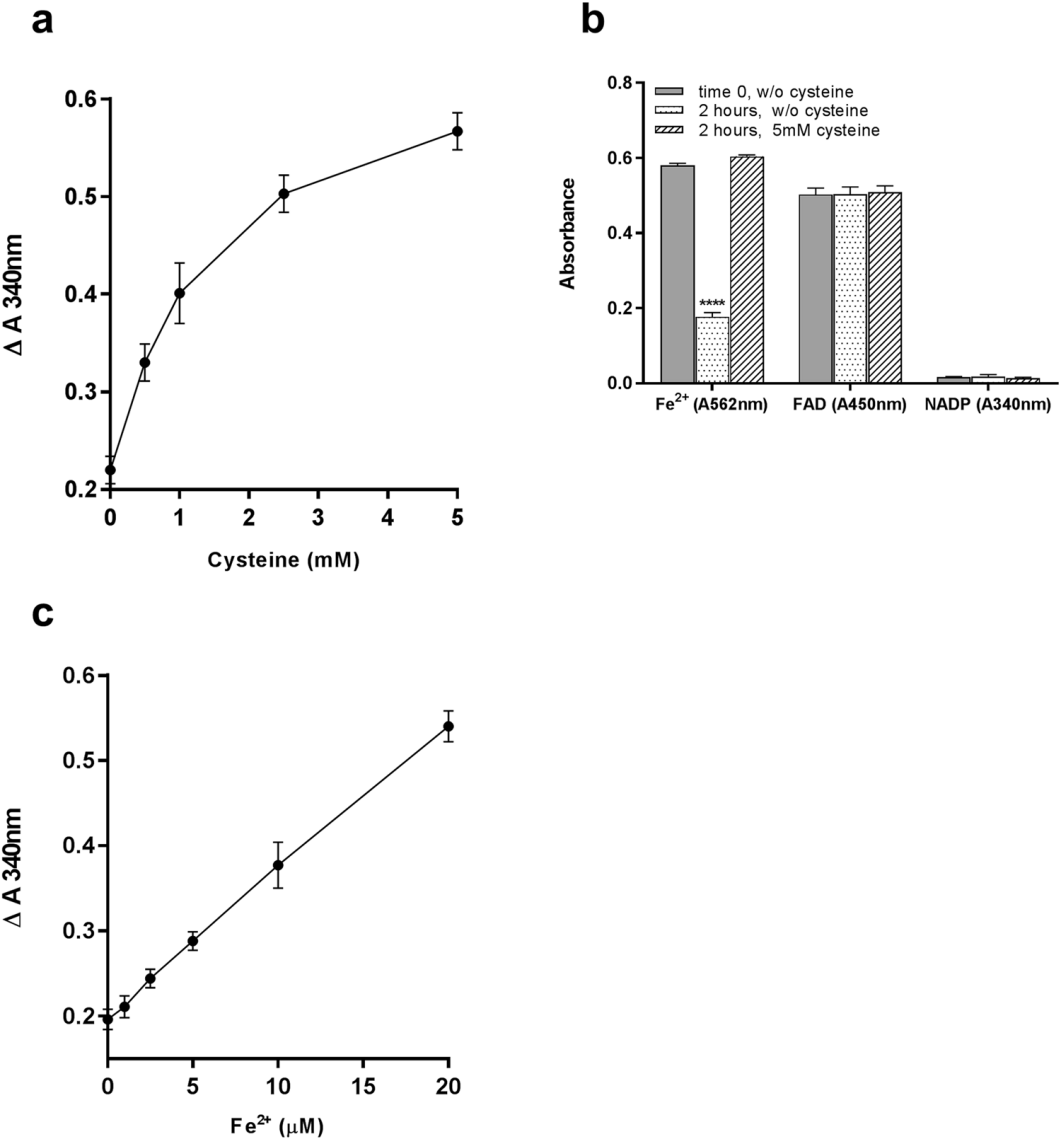
Participation of Fe^2+^ in FlaR enzymatic activity. (**a**) Cysteine significantly stimulates rFlaR NADP-reductase activity (Spearman correlation, r = 1, **p = 0.0083). (**b**) Solutions containing either 10 µM Fe^2+^ or 200 µM NADP or 50 µM FAD were incubated with cysteine. The oxidative state of Fe^2+^ was assessed with ferrozine staining and monitored at A_562_ nm. The oxidative state of FAD was monitored at A_450_ nm and that of NADP monitored at A_340_ nm. Fe^2+^, FAD and NADPH in aqueous solutions were allowed to oxidize for 2 hrs, and then cysteine was added for additional 2 hrs. Oxidation was observed only in Fe^2+^ solution which was significantly reduced upon cysteine addition. The results presented are the mean of three different experiments (one way ANOVA with Dunnett’s post hoc multiple comparisons test, n = 3 in each group, n = 3 in each group, ****p = 0.0001). (**c**) Increasing concentrations of Fe^2+^ significantly enhanced rFlaR NADP-reductase activity (Pearson correlation, r = 0.9947, ****p < 0.0001).

The next step was to determine the electron donor in rFlaR reductase activity. When cysteine was used as a reducing agent, the rFlaR FAD-dependent NADP reduction was enhanced in a concentration-dependent manner (Fig. 6a, Spearman correlation, r = 1, p = 0.0083). To detect which component in the reaction mixture was reduced cysteine was applied to solutions containing either ammonium iron sulfate, or FAD or NADP. Ammonium iron sulfate incubated for 2 hours at room temperature was found to be significantly oxidized (Fig. 6b, p = 0.0001). Addition of cysteine reduced the oxidized Fe^3+^ to Fe^2+^, as detected by the ferrozine stain (Fig. 6b; p = 0.0001). Both NADP and FAD were tested in their oxidized forms and no reduction in NADP and FAD could be observed in the presence of cysteine. These findings suggest that the stimulation of rFlaR activity occurs through the recycling of Fe^3+^ to Fe^2+^ by cysteine. To verify this point, excess Fe^2+^ ions were supplemented into the reaction mixture containing rFlaR, FAD and NADP. Indeed, rFlaR NADP reductase activity was stimulated in the presence of Fe^2+^ in a concentration-dependent manner (Fig. 6c, Pearson correlation, r = 0.9947, p < 0.0001).

### FlaR contributes *S*. *pneumoniae* resistance under oxidative stress conditions

**Figure 7:**
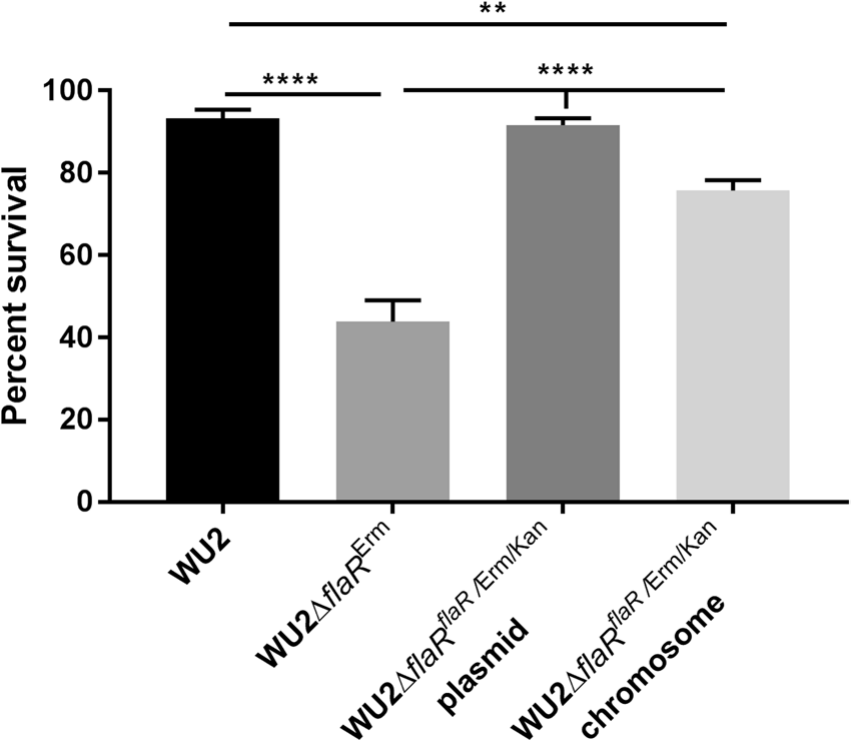
FlaR facilitates *S*. *pneumoniae* growth under oxidative stress. The WU2 WT, WU2Δ*flaR* ^Erm^ and complemented strains WU2Δ*flaR* ^*flaR*^/^Erm/Kan^ plasmid and WU2Δ*flaR* ^*flaR*/Erm/Kan^ chromosome were inoculated into 20 ml of THY at initial OD_620_ = 0.05 and grown to OD_620_ = 0.35 under anaerobic conditions. Then, bacteria were harvested, resuspended in PBS and incubated with equal volumes of either PBS (control) or H_2_O_2_, at final concentration of 20 mM. The WU2Δ*flaR* ^Erm^ mutant and, to a lesser extent, WU2Δ*flaR* ^*flaR*/Erm/Kan^ chromosome strain exhibited significant susceptibility to the oxidative stress, relative to the WU2 WT. The results presented are the mean of three different experiments, each performed in triplicates (one way ANOVA with Dunnett’s post hoc multiple comparisons test, n = 3 in each group, ** p = 0.0011; ****p = 0.0001).

To determine the importance of FlaR to *S*. *pneumoniae* resistance to oxidative stress, *flaR* null mutant (WU2Δ*flaR* ^Erm^) and complemented strains (WU2Δ*flaR* ^*flaR*/Erm/Kan^ plasmid and WU2Δ*flaR* ^*flaR*/Erm/Kan^ chromosome) were constructed as described in the Methods section. No differences in the growth rates could be observed when WU2 WT and all above strains were grown in THY under anaerobic conditions (Supplementary Fig. S3). Following a challenge with 20 mM H_2_O_2_, significantly reduced survival of WU2Δ*flaR* ^Erm^ was measured, 44%, compared to 93%, 92%, 76% survival of WU2 WT, WU2Δ*flaR* ^*flaR*/Erm/Kan^ plasmid and WU2Δ*flaR* ^*flaR*/Erm/Kan^ chromosome respectively, (Fig. 7, p = 0.0001). These findings highlights the involvement of FlaR’s in oxidative stress resistance. However, chromosomal complementation did not fully restore bacterial survival under oxidative stress conditions in comparison to the WT (Fig. 7, p = 0.0011).

### FlaR contributes to *S*. *pneumoniae* virulence

**Figure 8:**
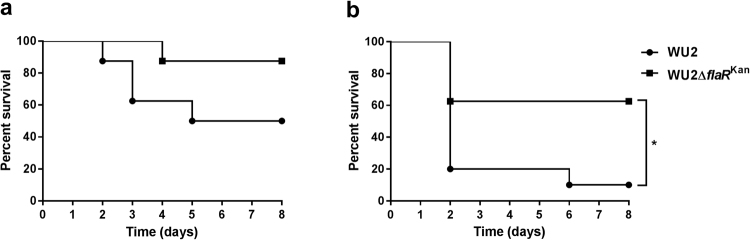
Reduced virulence of WU2Δ*flaR* ^Kan^ mutant. (**a**) Seven-week-old BALB/c mice were inoculated IP with 64 CFU of WU2 WT (n = 8) or 79 CFU of WU2Δ*flaR* ^Kan^ (n = 7) and survival was monitored daily (Log-rank (Mantel-Cox) test). (**b**) Seven-week-old CBA/N^*xid*^ mice were inoculated IP with 80 CFU of WU2 WT (n = 10) or WU2Δ*flaR* ^Kan^ (n = 8) and survival was monitored daily (Log-rank (Mantel-Cox) test *p = 0.0236).

To test whether FlaR contributes to *S*. *pneumoniae* virulence, BALB/c mice were inoculated intraperitoneally (IP) with WU2 WT or WU2Δ*fla*R^Kan^ strains. Increased survival was observed in mice inoculated with WU2Δ*fla*R^Kan^ compared to inoculation with the WT WU2, though it did not reach statistical significance (Fig. 8a). In an attempt to increase signal-to-noise responses, IP inoculation was performed using CBA/CaHN-Btkxid/J mice (CBA/N^*xid*^). This mouse strain carries a mutation in the Bruton kinase (*btk*) gene that renders it highly susceptible to *S*. *pneumoniae* infection^44,45^. Significantly increased survival rates were observed in CBA/N^*xid*^ mice inoculated IP with WU2Δ*fla*R^Kan^ compared to mice inoculated with WU2 WT (Fig. 8b, p = 0.0236).

### Mechanisms underlying FlaR contribution to *S*. *pneumoniae* virulence

**Figure 9:**
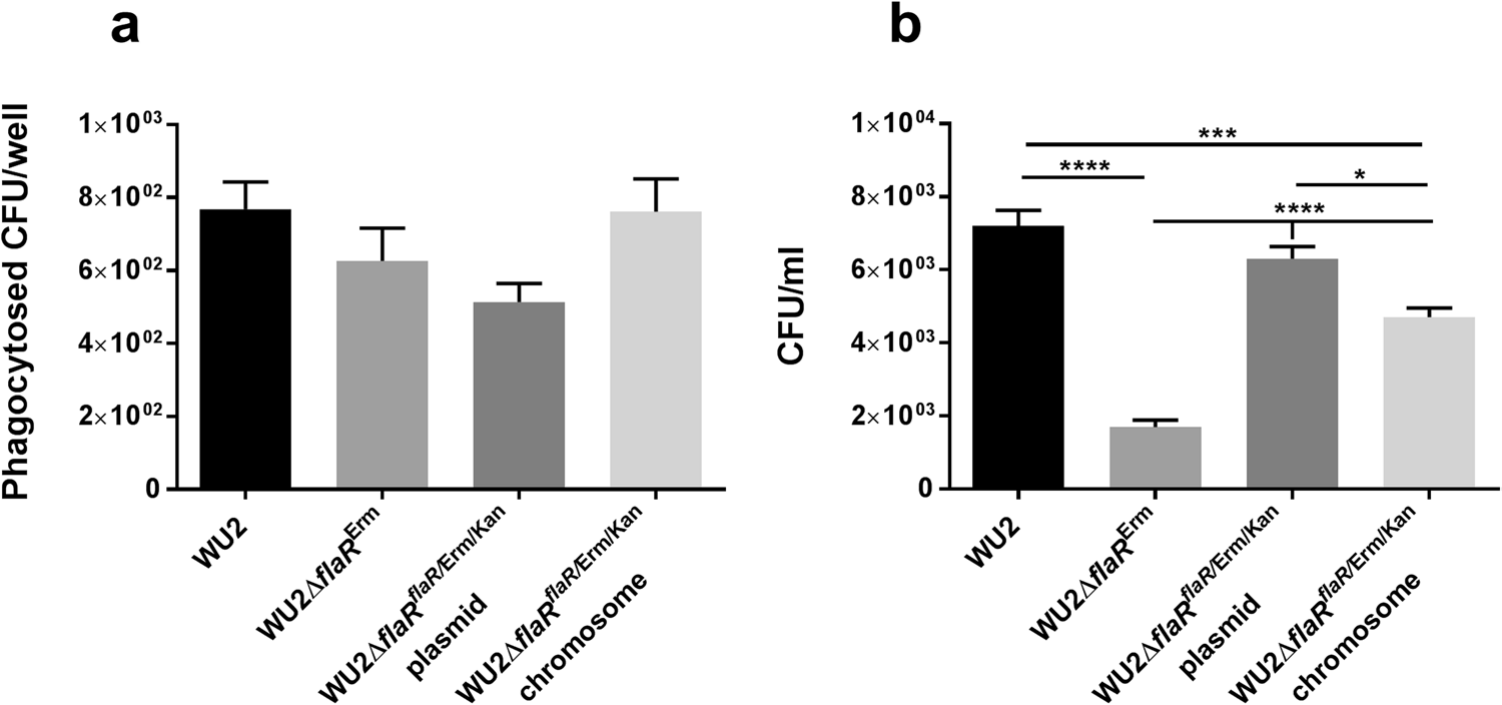
Mechanisms underlying FlaR contribution to *S*. *pneumoniae* virulence. (**a**) Phagocytosis assay. Mice peritoneal cells were plated onto 96 well culture plates (2 × 10^5^ cells/well). Adherent cells were incubated with either WU2 WT, WU2Δ*fla*R^Erm^ or WU2Δ*fla*R^*flaR*/Erm/Kan^ complemented strains (2.5 × 10^4^ CFU/well) for 40 min. Aliquots of the supernatant were plated onto blood agar plates for enumeration. The number of phagocytosed bacteria is presented as the difference between initial bacterial CFU/well and the residual live bacteria CFU/well. Presented here is the summary of 2 biological independent experiments each performed in triplicates (One-way ANOVA, p = 0.1190, non-significant). (**b**) FlaR mediates bacterial adhesion to host cells. The WU2 WT, WU2Δ*flaR* ^Erm^ and complemented WU2Δ*flaR* ^*flaR*^/^Erm/Kan^ plasmid and WU2Δ*flaR* ^*flaR*/Erm/Kan^ chromosome strains (MOI 10:1) were added to A549 cells, cultured in 96 well plates for 1 h at 37 °C. Excess bacteria were then removed, cells released with 0.25% trypsin-EDTA and plated onto blood agar plates for enumeration. The WU2Δ*flaR* ^Erm^ mutant and, to a lesser extent, WU2Δ*flaR* ^*flaR*/Erm/Kan^ chromosome strain exhibited significant inhibition of adhesion, relative to the WU2 WT and to the WU2Δ*flaR* ^*flaR*^/^Erm/Kan^ plasmid. The experiment was repeated three times on different occasions. Results presented here is of a representative experiment performed in quadruplicates (one way ANOVA with Dunnett’s post hoc multiple comparisons test, n = 4 in each group, *p = 0.0103; ***p < 0.0004; ****p = 0.0001).

The reduced virulence in the peritoneum in the absence of FlaR may result from two different mechanisms: increased sensitivity to oxidative stress elicited by immune cells or reduced adhesion and spread in the host. To test the first possibility, the extent of phagocytosis of WU2 WT, WU2Δ*fla*R^Erm^ and WU2Δ*fla*R^*flaR*/Erm/Kan^ complemented strains was evaluated. Interestingly, no significant differences in the phagocytosis by primary mice macrophages were observed for all of these strains (Fig. 9a, p = 0.1190). These data suggest that the reduced pneumococcal virulence in the peritoneum in the absence of FlaR is not due to increased sensitivity to oxidative stress produced by the recruited immune cells. This is in accordance with data published recently, showing that oxidation is not the major killing mechanism of *S*. *pneumoniae* by phagocytic cells^46^.

The surface expression of FlaR, presented in the previous^35^ and the current studies (Fig. 1), encouraged testing the second mechanism of FlaR involvement in virulence i.e adhesion. As demonstrated in Fig. 9b, WU2Δ*flaR* ^Erm^ mutant adhesion to lung-derived epithelial (A549) cells was significantly reduced compared to the WU2 WT (~76%; Fig. 9, p = 0.0001), WU2Δ*fla*R^*flaR*/Erm/Kan^ plasmid (~73%, p = 0.0001) and WU2Δ*fla*R^*flaR*/Erm/Kan^ chromosome (~64%, p = 0.0001). Complementation in WU2Δ*fla*R^*flaR*/Erm/Kan^ plasmid restored bacterial adhesion to A549 cells to about 88%, not significantly differing from the WU2 WT. The adhesion of WU2Δ*fla*R^*flaR*/Erm/Kan^ chromosome strain was restored only up to 65%, remaining significantly lower compared to the WU2 WT (Fig. 9b, p = 0.0011). These data suggest that FlaR is involved in bacterial adhesion to the host cells in addition to its enzymatic function. Similarly, reduced adhesion to the glioblastoma cell line U251^47^ and the motor neuron cell line NSC 34^48^ was observed with WU2Δ*flaR* ^Erm^ in comparison to the WT and to the two complemented strains (data not shown).

### Vaccine potential of rFlaR

The finding that FlaR contributes to *S*. *pneumoniae* virulence, combined with the lack of significant similarity to any human protein, directed us to test the vaccine potential of rFlaR. Immunization of BALB/c mice with rFlaR elicited the production anti- rFlaR antibodies. An increase in antibody levels could be observed following the secondary immunization however this increase reached significance only after the tertiary immunization. The antisera titer following the third immunization reached ~1:10000 (Supplementary Fig S4, p = 0.014).

**Figure 10:**
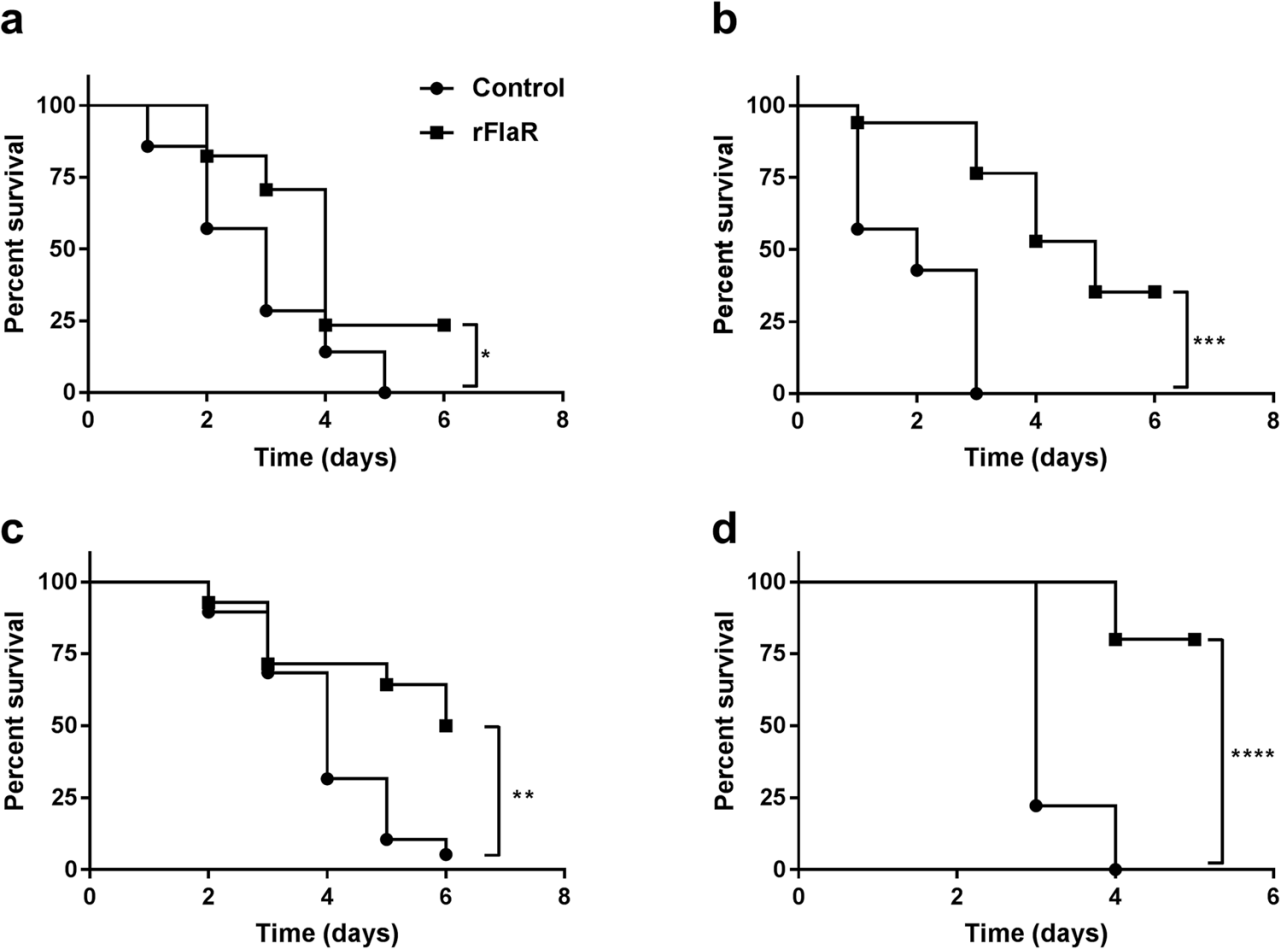
Vaccine potential of rFlaR. Immunization of BALB/c mice with rFlaR conferred significant protection against IP lethal *S*. *pneumoniae* challenge. (**a**) 15 µg (Log-rank (Mantel-Cox) test, n = 7 in the control and n = 17 in the immunized group, *p = 0.0388) and (**b**) 25 µg rFlaR in the presence of alum as an adjuvant and (Log-rank (Mantel-Cox) test, n = 7 in the control and n = 17 in the immunized group,***p = 0.0001) (**c**). 35 µg rFlaR using CFA in primary immunization and IFA in booster immunizations (Log-rank (Mantel-Cox) test, n = 19 in the control and n = 14 in the immunized group,**p = 0.0053). (**d**) BALB/c mice inoculated IP with WU2 WT treated with anti-rFlaR antiserum obtained from immunized rabbits survived significantly longer then mice inoculated with WU2 WT treated *ex-vivo* with preimmune serum (Log-rank (Mantel-Cox) test, n = 10 in the control and n = 10 in the immunized group, ****p < 0.0001).

In addition, significant protection against IP lethal *S*. *pneumoniae* challenge was observed following immunization with rFlaR in comparison to adjuvant only immunization (Fig. 10a 15 µg rFlaR + alum, p = 0.0388; Fig. 10b 25 µg rFlaR + alum, p = 0.0001; Fig. 10c 35 µg rFlaR + CFA/IFA, p = 0.0053). To further demonstrate rFlaR ability to elicit protective immune response we have performed a modified method of passive immunization. Bacteria were incubated *ex-vivo* with either pre-immune or anti-rFlaR antiserum. Mice were inoculated IP with bacteria pre-treated with the anti-rFlaR antiserum survived significantly longer than mice inoculated with bacteria pre-treated with pre-immune serum (Fig. 10d, p < 0.0001).

## Discussion

A putative flavin reductase (FlaR) was recently annotated in *S*. *pneumoniae* TIGR4 strain in the NCBI database. We have previously shown that there is an age-dependent enhancement of the antibody response to this protein^35^. Therefore, we initially named it pneumococcal surface immunogenic protein B (PsipB), but in order to conform with the NCBI database we currently refer to this protein as FlaR. FlaR was found to be surface expressed, although it is devoid of a signal peptide or any other known export sequences^35^. Several additional *S*. *pneumoniae* proteins such as α-enolase^49,50^, glyceraldehyde 3-phosphate dehydrogenase, fructose-bisphosphate aldolase^35^, glutamyl tRNA synthetase^51^, NOX^52^ and PtsA^39^ were also shown to be cell surface localized albeit lack of known export signals^53,54^. The current study further confirmed the cell surface localization of FlaR, using immunoblotting and flow cytometry.

FlaR was found to be highly conserved in different *S*. *pneumoniae* strains. In addition, highly conserved cysteines at positions 66, 72 and 84 of WP_000580663.1 were found in all *S*. *pneumoniae* sequenced flavin reductases. Multiple cysteines in a protein may enable the formation of multimers. Indeed, resolving rFlaR protein on SDS-PAGE demonstrated the existence of both dimeric and monomeric forms of FlaR. Equilibrium between the dimeric and monomeric forms may be important for FlaR enzymatic activity, as hydrogenases are known to often function as homo-dimers^55^ or multi-enzyme complexes^56^. Moreover, multiple cysteines in a protein sequence may indicate a transitional metal ion binding capacity^57^. Metal ions can interact with metal-binding proteins during their folding and create a local structure that initiates and directs the protein-folding process and is considered critical for its biological activity^58,59^. In accordance, rFlaR could be refolded/solubilized at physiological pH only in the presence of Fe^2+^ and to lesser extent with Co^2+^ and Cu^2+^. Noteworthy is the finding that rFlaR, refolded at pH 8 without Fe^2+^, bound Fe^2+^ as detected by the ferrozine stain.

We validated the putative flavin reductase activity of rFlaR and found that NADP and FAD are necessary components for its enzymatic activity, as described for NADPH reductases^60^. In contrast, the flavin reductase activity of ferredoxin-NADP^+^ reductase from *Pseudomonas putida* was FAD-independent^42^. The activity of NADPH-cytochrome p 450 reductase is generally accompanied by measurable alterations in FAD and FMA oxidative state^60^. Although rFlaR catalyzed NADP reduction in a FAD-dependent manner, we failed to detect the change in FAD oxidative state.

Refolding of rFlaR in the presence of Fe^2+^ enhanced its enzymatic activity in comparison to the rFlaR refolded at pH 8, possibly as a result of increased initial binding of Fe^2+^. The ability of divalent ion chelators to inhibit rFlaR activity reinforce Fe^2+^ necessity for NADP reduction by FlaR.

The midpoint potential of Fe^2+^ in solution, as a complex with EDTA, is +0.1 V while the midpoint potential of NADP is much lower (−0.32 V), rendering reduction of NADP by Fe^2+^ improbable. However, the midpoint potential of Fe^2+^ bound to 4Fe-4S cluster of ferredoxin from *Butyribacterium methylotrophicum* was found to be −0.410 V^61^ and −0.472 V in 2Fe-2S cluster of oxidoreductase from *Paracoccus denitrificans* ^62^. Additionally, it was shown that substitution of amino acid residues around an Fe^2+^-sulfur cluster, by site-directed mutagenesis, affects the midpoint potential of the Fe^2+^ in the cluster^63^. The finding that under physiological conditions rFlaR remains soluble only in the presence of Fe^2+^, impedes determination of the midpoint potential of Fe^2+^ bound to FlaR. Yet, the demonstration of Fe^2+^ dependent rFlaR NADP reductase activity indicates that the FlaR-bound Fe^2+^ midpoint potential is probably lower than that of NADP.

Mucosal surfaces lack free Fe^2+^ due to chelation by host Fe^2+^ -binding proteins^64^. *S*. *pneumoniae* has several mechanisms enabling it to cope with iron deficiency, among them hijacking iron from hemin, hemoglobin or lactoferrin^33,65^, or direct iron binding by ABC iron transporters Pit1B and Pit2A^31^. A double mutant of *pit1B* and *pit2A* demonstrated growth deficiency in a cation-depleted medium, which was restored by the addition of Fe^2+^ ^31^. In addition, the 12 mer Dpr/Dps that bind, oxidize and store ~500 Fe^2+^ atoms in its hollow cavity has been identified in streptococci^27,66^. The current study revealed that FlaR belongs to the Fe^2+^ binding proteins.

rFlaR activity was found to be enhanced in the presence of increasing concentration of the reducing agent, cysteine. Our demonstration that cysteine reduced Fe^3+^ but not FAD or NADP, implies that the increase in NADPH in the presence of cysteine stemmed from Fe^3+^ to Fe^2+^ recycling, which stimulated rFlaR activity. In line is the finding that rFlaR NADP reductase activity was enhanced in a Fe^2+^ concentration-dependent manner.

Tight regulation of the free Fe^2+^ pool can be especially important for pneumococci, which produce high levels of H_2_O_2_ but lack catalase, and hence are at high risk of damage by ROS formed via the Fenton reaction^13^. We hypothesized that FlaR is involved in preventing the accumulation of toxic levels of free Fe^2+^ that may react with H_2_O_2_ and produce ROS. Indeed, WU2Δ*flaR* demonstrated marked susceptibility to H_2_O_2_ and complementation restored bacterial resistance to H_2_O_2_. Of note, the strain complemented with the plasmid demonstrated increased resistance to H_2_O_2_ in comparison to the strain complemented in chromosome. The explanation may be that following *flaR* trans-complementation, multi-copies of *flaR* are expressed, whereas following cis-complementation (into the same chromosome) a single copy of *flaR* is expressed, under the control of its native promoter. This may stem from two concomitantly occurring mechanisms: i) FlaR’s function in binding and oxidizing Fe^2+^ to protect the bacterium from the toxic effects of the Fenton reaction; ii) FlaR’s NADP reductase activity provides an additional source of NAD(P)H beside the Embden-Meyerhof-Parnas and the pentose phosphate pathways. Increased NAD(P)H levels are essential under high H_2_O_2_ levels for NADH oxidase activity^67^. The action of NADH oxidase makes pyruvate available for SpxB oxidation, resulting in the production of two additional ATP molecules per one glucose molecule, beyond that available from conventional glycolysis^13,68^. Moreover, the electron transport to NADPH by FlaR enzymatic activity may increase the reduction of cysteine containing enzymes^69^ to maintain redox balance thus enhancing bacterial growth and virulence^70^.

In contrast to our finding that the presence of FlaR in the cells confer resistance to oxidative stress, Pericone *et al*.^13^ claimed that iron chelators had no effect on the survival of WT or a mutant lacking pyruvate oxidase (Δ*spxB*) following a challenge with 20 mM H_2_O_2_. This led the authors to suggest that the Fenton reaction does not play a major role in the killing of *S*. *pneumoniae* by H_2_O_2_. The mechanism by which FlaR contributes to bacterial survival under oxidative stress conditions should be further elucidated. Of note is the finding that Fe^2+^ dependent-rFlaR activity was not affected by the H_2_O_2_ scavenger, salicylate, suggesting that Fe^2+^ oxidation is not directly executed by H_2_O_2_.

The contribution of FlaR to pneumococcal virulence was tested in a mouse model of sepsis. The reduced virulence of *S*. *pneumoniae* in the peritoneum, in the absence of FlaR, could be explained by increased sensitivity to phagocytosis and oxidative stress elicited by immune cells. The phagocytic role of macrophages in *S*. *pneumoniae* engulfment and killing has been previously demonstrated for bronchoalveolar lavage derived macrophages^71^, peritoneal macrophages^72^ and neutrophils^46,73^. Interestingly, we found that FlaR absence did not significantly affect the extent of phagocytosis by primary mice peritoneal macrophages. In line, *in vitro* studies demonstrated that the predominant killing mechanism of pneumococci in the phagosome of neutrophils is executed by the proteolytic activity of two serine proteases rather than oxidation^46,73^. The involvement of the serine proteases in pneumococcal killing was further confirmed *in vivo* demonstrating higher bacterial load in the spleen of mice lacking the serine proteases in a systemic infection model^46^. These authors also emphasized that in the case of systemic infection with *S*. *pneumoniae*, most of the bacteria can be found in the spleen and to a lesser extent in other organs^46^.

The peritoneum is characterized by low oxygen pressure^74^. Thus, the role of FlaR in protection against oxidative stress could not explain the reduced virulence of WU2Δ*flaR* ^kan^ in this niche suggesting an additional role for FlaR in virulence. FlaR was found to be surface expressed using immunoblotting of cell-wall proteins and flow cytometry. Pre-incubation of *S*. *pneumoniae* with anti-FlaR antisera, which interacts only with the surface exposed FlaR, reduced bacterial virulence, reinforcing the surface localization of FlaR. Many surface proteins were found to mediate bacterial adhesion to the host^37^. Hence, we hypothesized that FlaR may be involved in bacterial adhesion to host cells. Indeed, WU2Δ*flaR* ^Erm^ strain was found to adhere to A549 cells significantly less than its parental WT and the two complemented strains. We have also used glioblastoma cells U251^47^ and motor neuron cells NSC34^48^ to establish FlaR contribution to *S*. *pneumoniae* adhesion and similar results were obtained. The ability of anti-rFlaR antibodies to reduce bacterial virulence also implies a direct involvement of the surface expressed FlaR in virulence and diminishes the possibility that downstream consequences of the mutation, such as upregulation of other adhesins, are responsible for the bacterial virulence.

The following findings highlight FlaR as a potential candidate vaccine: 1) possesses age dependent antigenicity; 2) is conserved among *S*. *pneumoniae* strains; 3) lacks significant homology to human proteins; 4) protects the bacterium against oxidative stress and 5) possibly mediates *S*. *pneumoniae* adhesion to the host. Indeed, FlaR elicited significant protection against IP challenge. In addition, sera obtained from immunized rabbits were able to neutralize *S*. *pneumoniae* virulence in the peritoneum, probably by enhancing opsonophagocytosis and/or interfering with bacterial adhesion and spread. Hence, rFlaR can be considered as a future vaccine candidate. It should be noted that proteins, such as PspA, CbpA, PavA, PavB and PhtD, which are involved in different aspects of bacterial virulence including adhesion, are known to be immunogenic and to elicit a protective immune response in experimental infection models^75–79^. Furthermore, PhtD has recently been shown to elicit an immune response in phase I/II clinical trials^80,81^.

In summary, this study attributes FlaR multiple functions in *S*. *pneumoniae* physiology and virulence: i) Fe^2+^ binding and oxidizing to prevent the toxic effect of the Fenton reaction; ii) reducing NADP to provides an additional source of NAD(P)H, resulting in maintenance of redox balance and ATP production; and iii) mediating bacterial adhesion to the host. Importantly, we found that rFlaR can be considered for future vaccine development. Further studies should concentrate on detailed understanding of FlaR’s role in pneumococcal biology and the nature of the immune protection elicited by FlaR.

## Methods

### Inoculation and immunization of mice and ethics statements

This study was carried out in strict accordance with the recommendations in the Guide for the Care and Use of Laboratory Animals of the National Institutes of Health. The protocol was approved by the Institutional Animal Care and Use Committee of the Ben-Gurion University of the Negev, Beer Sheva, Israel (Permit number: 53.08.08).

Seven-week-old BALB/cOlaHsd (BALB/c) female mice (Harlan Laboratories, Israel) or seven-week-old CBA/CaHN-Btk^*xid*^ (CBA/N^*xid*^; Jackson Laboratories, Bar Harbor, ME, USA) mice were housed in sterile conditions under 12-h light/dark cycles and fed Purina Chow and tap water ad libitum. The groups were matched for age.

BALB/c mice were inoculated IP (without anesthesia) with the WU2 WT (n = 8; 64 CFU) or WU2Δ*flaR* ^Kan^ (n = 7; 64 CFU) strains. Survival was monitored daily. Additionally, CBA/N^*xid*^ mice were inoculated IP (without anesthesia) with WU2 WT (n = 10; 80 CFU) or WU2Δ*flaR* ^Kan^ (n = 8; 80 CFU), and survival was monitored daily.

BALB/c mice were immunized subcutaneously with 15 µg rFlaR (rFlaR + alum n = 17; alum n = 7) or 25 µg rFlaR (rFlaR + alum n = 17; alum n = 7) mixed with 75 µl of the Imject Alum adjuvant (Pierce Biotechnology, Inc., Rockford, IL) or 35 µg rFlaR emulsified with complete Freud’s adjuvant (CFA) on primary immunization and boosted (days 14 and 28) with rFlaR emulsified with incomplete Freud’s adjuvant (IFA) (rFlaR + CFA/IFA n = 19; CFA/IFA only, n = 14). Mice were challenged IP on day 42, under deep anesthesia using isoflurane (Piramal Critical Care Inc., PA, USA) with a lethal dose of WU2 WT strain (~10^2^ CFU).

*Ex-vivo* neutralization was performed as follows: a lethal dose (~10^2^ CFU) of WU2 WT strain was incubated at 37 °C for 1 hour with 1:10 diluted rabbit pre-immune serum or anti-rFlaR serum and subsequently inoculated IP to BALB/c mice (n = 10 in each group). Survival was monitored daily.

For all animal experiments, mice were humanely euthanized by CO_2_ asphyxiation, as recommended by the American Veterinary Medical Association (AVMA) guidelines for euthanasia 2013, if they became moribund or showed evidence of distress. The following criteria were considered sufficient evidence of distress to warrant such intervention in order to minimize pain and suffering to animals: severe weight loss (20% body weight); reluctance or inability to move freely; appearance of bristle fur; social disengagement; refusal or inability to eat or drink. No analgesic treatment was provided as such treatment may alter the immune response and may independently affect the outcome of the experiments^82^.

### Chemicals and Biological Reagents

Unless otherwise stated, all chemicals and biochemicals of highest purity available were purchased from Sigma-Aldrich Corp. (St Louis, MO). FAD and NADP were purchased from Applicheme GmbH (Darmstadt, Germany). For research involving biohazards, correct standard procedures have been carried out.

### Bacterial Strains and Growth Media

*S*. *pneumoniae* serotype 3 strain WU2^44^ and its derivatives, described below, were used. Pneumococci were grown on blood agar plates or in Todd-Hewitt broth supplemented with 0.5% (w/v) yeast extract (THY). Two *Escherichia coli* strains, DH5α UltraMAX (DH5α; Invitrogen Corp, Carlsbad, CA, USA) and *E*. *coli* BL21(DE3)pLysS (BL21; Promega Corp, Madison, WI), were grown in Luria broth (LB), Lennox.

### Cloning, Expression and Purification of rFlaR, rFabD, rATCase and antibodies production

The *flaR* gene was amplified from *S*. *pneumoniae* strain WU2 genomic DNA by PCR using the primers FlaR-F and FlaR-R (Supplementary Table S1, *flaR* pHAT expression) designed according to the sequence in the R6 strain (spr0489). The amplified and *Bam*HI-*Sac*I (Takara Bio Inc, Shiga, Japan)-digested DNA-fragments were cloned into the pHAT expression vector (BD Biosciences Clontech, Palo Alto, CA) and transformed into DH5α *E*. *coli* cells. The vector was purified using the Qiagen High Speed Plasmid Maxi Kit (Qiagen GMBH, Hilden, Germany) and transformed into *E*. *coli* BL21. Sequencing was performed to rule out any mutation. rFlaR expression was induced by incubation for 5 h with 1 mM of IPTG at 37 °C. The protein was purified under denaturing conditions using Ni-NTA agarose beads (Qiagen GMBH). This preparation was dialyzed against PBS pH 7.3, containing 2 mM FeSO_4_ for 48 h with three changes of buffer (named rFlaR Fe^2+^). Isolation of the protein was confirmed by immunoblotting with anti-HAT (BD Biosciences Clontech) and thereafter with anti-rFlaR antibodies (described below) and trypsin in-gel digestion of the protein to derive peptides for MALDI-TOF mass spectrometry sequencing (Bruker Reflex-IV mass spectrometer Bruker-Daltonik, Bremen, Germany). Alignment to existing databases was performed using both the Mascot software package (Matrix Science Ltd., UK, http://www.matrixscience.com) and the “Profound” Program provided by the Rockefeller University.

Refolding of rFlaR was performed using 20 mM of the following buffers: Acetate pH 5.0, 2-(N-morpholino)ethanesulfonic acid (MES), pH 6.0, phosphate pH 7.0 and pH 8.0, tris(hydroxymethyl)aminomethane pH 9.

Untagged protein was produced by amplifying the gene using the *S*. *pneumoniae* TIGR4 strain (ATCC, Rockville, MD, USA) DNA as template, using specific primers (Supplementary Table S1, *flaR* pET30a (+)). Digestion of pET30a(+) (Novagen, Madison, WI, USA) with *NdeI* and *Bpu1102I* restriction enzymes, prior to ligation removed all tags from the vector. Then, *flaR* was cloned into the vector. Protein expression was induced with IPTG in fermented *E*. *coli* BL21 followed by inclusion bodies isolation. The inclusion bodies were centrifuged and resuspended in double distilled water and frozen in aliquots. The inclusion bodies were refolded by dialysis for 1 hour against a buffer adjusted to pH 11.3, containing 4.5 M urea, 40 mM Tris base and 1 mM cysteine, followed by the addition of 0.67 mM arginine and continued dialysis overnight. The resulting solution was concentrated ~4.4 fold. This preparation was applied to a preparative Superdex 200 column and elution was performed with buffer adjusted to pH 10, containing 4.5 M urea, 40 mM Tris base and 1 mM cysteine. The tubes containing the protein were pooled and dialyzed against NaHCO_3_ at pH 11 followed by lyophilization. The lyophilized protein was suspended in PBS pH 8 (named FlaR pH 8).

For antibodies production, three-month-old New Zealand white rabbits and 7-week old BALB/c mice were immunized with Ni-NTA-purified rFlaR formulated with CFA in the first immunization and IFA in the two booster immunization with two-week intervals between immunizations. The rabbits were bled following the third immunization.

FabD was cloned and expressed as a His-tagged soluble protein and antibodies were produced as previously described^36^.

ATCase is known to be involved in pyrimidine synthesis. ATCase is a cytoplasmic protein that was found to be surface exposed^37^. *ATCase* (SP_RS06260) was cloned and expressed as a His-tag soluble protein. Briefly, *ATCase* was amplified from the R6 strains using specific primers (Supplementary Table 1, *ACTaase* gene for pET32a+) and ligated into pET32a+ (Novagen, Madison, WI, USA) following digestion with EcoR1 and Xho1 restriction enzymes. The plasmid was transformed into DH5α *E*. *coli* cells. The vector was purified and transformed into *E*. *coli* BL21. Sequencing was performed to rule out any mutation. Protein expression was induced by 0.1 mM IPTG for 3 h. Following bacterial lysis the protein was purified using NI-NTA column under native condition.

### Flow cytometry of *S*. *pneumonia*

Flow cytometry was performed as previously described^51^. Briefly, R6 bacteria were incubated with mouse anti-rFlaR antibodies or control mouse serum, washed, and stained with Alexa Fluor 647^®^-conjugated goat-anti-mouse-IgG (Jackson ImmunoResearch, West Grove, PA). Flow cytometry was performed using a FACSCalibur flow cytometer (Becton Dickinson, Mountain View, CA), and data were acquired and analyzed using BD CellQuest^TM^ 3.3 software.

### Isolation of *S*. *pneumoniae* Cell-Wall (CW) Proteins

CW proteins were isolated by the method of Siegel *et al*.^83^. Briefly, bacteria were harvested by centrifugation at 4700 *g* for 15 min, washed with PBS and incubated with 3 ml protoplast buffer (20% sucrose, 2.5 mM MgCl_2_, 5 mM Tris-Cl, pH 7.4), 1000U mutanolysin and 0.2 ml protease inhibitor cocktail for 1 h at 37 °C. The soluble proteins released from the cell-wall were collected after centrifugation at 25,000 g and stored at −70 °C.

### Immunobloting Analysis

Protein mixtures were separated by SDS-PAGE and transferred to nitrocellulose membranes (Bio-Rad Laboratories, Inc., Carlsbad, CA), according to manufacturer’s instructions. Immunobloting analysis of FlaR expression was performed with rabbit anti-sera obtained against purified rFlaR and detected with Cy™3 AffiniPure Donkey Anti-Mouse IgG (H + L) (Jackson ImmunoResearch, West Grove, PA).

### rFlaR NADP Reductase Assay

The reaction mixture contained 50 µM FAD, 200 µM NADP, 10 µM ammonium iron sulfate and 6 µM rFlaR in PBS buffer. NADP reduction was detected by increased absorbance at A_340_ nm, which does not overlap ferrous absorbance at A_240_ nm, as measured with a BioRad SmartSpec 3000 Spectrophotometer (Bio Rad; Oceanside, California, USA).

### Detection of Iron Binding

The assay was performed as described by Da Lozzo *et al*.^84^ with slight modifications. Briefly, rFlaR (6 µM) was mixed with PBS containing 20 µM ammonium iron sulfate. Ferrozine (50 µM) was added to the reaction mixture and absorbance at A_562_ nm was measured after 10 min. BSA (6 µM), rACTase and EDTA (10 nM) were used as negative and positive controls, respectively.

### Ability of Cysteine to Reduce Iron

Freshly prepared solution of 20 µM ammonium iron sulfate in PBS was incubated for 2 h at 25 °C to allow Fe^2+^ oxidation prior to the addition of 5 mM cysteine. The extent of Fe^2+^ oxidation in PBS solution was tested with ferrozine stain, and absorbance monitored at A_562_ nm. The same manipulations were performed for 200 µM NADP and 50 µM FAD solutions in PBS, and absorbance was measured at A_340_ nm and at A_450_ nm, respectively.

### Bioinformatics Analyses

All completely sequenced genomes of *S*. *pneumoniae* were downloaded from NCBI’s Genome database (29 genomes as of October 2^nd^, 2016). The flavin reductase amino acid sequence of the TIGR4 strain (WP_000580663.1) was used as a query in tblastn and blastp searches against each of these genomes and their predicted proteins. WP_000580663.1 sequence was then used as a query in a blastp search against all RefSeq proteins annotated as *S*. *pneumoniae* and “flavin reductase”. The 63 retrieved protein hits were subjected to multiple sequence alignment using Tcoffee with default parameters. A representative set of 9 divergent proteins was selected from the multiple sequence alignment by inspecting a Neighbor Joining tree and choosing a representative protein from each clade. Multiple sequence alignment of these proteins was performed using Tcoffee and visualized using the Jalview software. Finally, the entire RefSeq protein database except for *S*. *pneumonia* proteins was searched by blastp with WP_000580663.1 as a query. Only Genbank records containing the word “Flavin” and having blastp e value < 0.001 were retrieved.

### Preparation of WU2Δ*flaR* Mutant Bacteria (kanamycin or erythromycin resistance)

To create null mutants of the *flaR* gene, the kanamycin (Kan) or erythromycin (Erm) resistance cassette was inserted into the coding sequence of *flaR* via homologous recombination. The transformation procedure was performed as previously described^85^ with minor modifications. The upstream and downstream flanking regions of *flaR* were amplified by PCR from DNA of WU2 strain using the primer combinations upwing-F/upwing-R (Supplementary Table S1, Upwing WU2Δ*flaR*), for the upstream region, and downwing-F/downwing-R, for the downstream region (Supplementary Table S1, Downwing WU2Δ*flaR*). The upwing 1149 bp harbors 125 bp of *flaR*. The downwing 582 bp harbors 68 bp of the *flaR*. Hence, the expected length of the *flaR* fragment to be deleted was 277 bp. The Kan cassette was amplified by PCR from the genome of CP1250^86^ using the primer combinations Kan AB-F/Kan AB-R (Supplementary Table S1, Kan AB cassette). The Erm cassette was amplified by PCR from the genome of WU2Δ*nox* ^*Erm*52^ using the primer combinations Erm AM-F/Erm AM-R (Supplementary Table S1, Erm AM cassette). The PCR products were digested with the corresponding restriction nucleases, as specified in Supplementary Table S1, purified, ligated and transformed into WU2 in the presence of competence stimulating factor, CSP1 and CaCl_2_. Transformants were selected on THY solidified with 1.5% agar, containing kanamycin (80 µg/ml) or erythromycin (125 µg/ml). Verification of *flaR* deletion was done by PCR using specific primers (Supplementary Table S1, Kan AB cassette for Kan resistance and Erm AM cassette for Erm resistance). It is worth mentioning that phenotypically, *flaR* mutation did not affect the size and mucosity of the colonies.

Of note, the kanamycin resistant mutant strain, WU2Δ*flaR* ^Kan^, was used in the mice inoculation studies. The erythromycin resistant mutant strain, WU2Δ*flaR* ^Erm^, was used in the susceptibility to oxidative stress experiment and the adhesion to A549 cells assay.

### Construction of two WU2Δ*flaR*^*flaR*/Erm/Kan^ complemented strains

####  Trans-complementation of WU2Δ*flaR*^Erm^

Complementation was performed using Gram positive compatible plasmid, pBAV1K-T5-gfp (Addgene; Cambridge, MA), bearing the kanamycin resistance cassette. The *flaR* gene was amplified from WU2 genome using *flaR*- F and *flaR*-R primers (Supplementary Table S1, *flaR* pBAV-K1- plasmid and pCEP complementation), which introduce *NcoI* and *PstI* sites. Subsequently the plasmid was digested, resulting in *gfp* gene deletion and retaining the constitutive T5 viral promoter. Following the ligation of *flaR* to pBAV1K-T5-gfp, the plasmid was transformed into *E*. *coli* DH5α, overexpressed, purified and transformed into WU2Δ*flaR* ^Erm^ to produce WU2Δ*flaR* ^*flaR*/Erm/Kan^ complemented strain. Successful insertion of pBAV1K-T5^*flaR*^ was verified by PCR.

#### *Cis*-complementation of WU2Δ*flaR*^Erm^

To confirm that mutation of *flaR* in WU2Δ*flaR* ^Erm^ introduced no polar effects, WU2Δ*flaR* ^Erm^ was complemented with an intact copy of the gene using pCEP, which is a nonreplicative plasmid that allows controlled gene expression under its native promoter, following ectopic integration into the chromosome^87,88^. Briefly, *flaR* was amplified from WU2 with *flaR*- F and *flaR*-R primers (Supplementary Table S1, *flaR pBAV-K1-* plasmid and pCEP complementation), which introduce *NcoI* and *PstI* sites. Then the amplicons were ligated into *NcoI* and *PstI* digested vector. An aliquot of ligation mixture was transformed into Stellar™ competent cells (Clonetech, Saint-Germain-en-Laye, France) as described by the manufacturer. The transformants were selected for kanamycin resistance (500 μg/ml). Successful ligation was determined by colony PCR using Mal-F and pCEP-R primers (Supplemental Table S1, *flaR* pCEP verification), whose recognition sites are localized immediately up and downstream of the cloning site, respectively. These primers amplified 263 bp product in empty vector, while they produce a product of 913 bp in the recombinant clones (additional 650 bp represents the cloned fragment containing *flaR*). The recombinant plasmid was purified using a commercial kit (Qiagen), and an aliquot was transformed into WU2Δ*flaR* ^Erm^ as described previously^89^ to produce the complemented strain WU2Δ*flaR* ^*flaR*/Erm/Kan^ chromosome. The transformants were selected on blood agar plates supplemented with erythromycin (125 μg/ml) and kanamycin (500 μg/ml). Integration of *flaR* into the genome was confirmed by PCR (Supplementary Table S1, Kan resistance in pCEP verification).

### Oxidative stress assay

Oxidative stress assays were performed as previously described^87^ with minor modifications. Briefly, WU2 WT, WU2Δ*flaR* ^Erm^ and WU2Δ*flaR* ^*flaR*/Erm/Kan^ plasmid and chromosome strains were grown under anaerobic conditions in 20 ml of THY medium to an OD_620_ = 0.3. Five ml of culture were harvested by centrifugation at 4000 rpm for 20 min and resuspended in 5 ml of PBS. Even volumes of the bacteria were dispensed, mixed with equal volumes of either H_2_O_2_ (final concentration 20 mM) or PBS and incubated at 37 °C for 30 min. Following the incubation serial dilutions were plated onto blood agar plates for bacterial enumeration. The percent survival was calculated by dividing the CFU of cultures after exposure to H_2_O_2_ by the CFU in the control culture without H_2_O_2_ of the respective strain. The results presented are the average of three independent experiments performed in triplicates.

### Primary macrophage phagocytosis assay

Mice were injected intraperitoneally with 3% thioglycolate broth. Three days later mice were euthanized, using CO_2_, and their peritoneal cells were harvested by flushing the peritoneum with 10 ml of PBS. Cells were centrifuged and resuspended in complete (c) RPMI 1640 medium supplemented with 10% FCS and 2% L-glutamine. 2 × 10^5^ cells/well were plated onto 96 well culture plates. The macrophages were allowed to adhere for 9 hours and the supernatant was removed. 2.5 × 10^4^ of midlog harvested bacteria in RPMI 1640 were added to the well for 40 minutes incubation at 37 °C. Following the incubation, aliquots were taken from the supernatant and plated onto blood agar plates for enumeration. The number of residual live bacteria CFU was subtracted from the initial bacterial CFU resulting in the number of phagocytosed bacteria. The data presented is the summary of 2 biological independent experiments each performed in triplicates.

### *S*. *pneumoniae* adhesion to A549 cells assay

A549 cells (lung adenocarcinoma cells; ATCC, Rockville, MD, USA) retain morphological, biochemical and immunological characteristics resembling type II lung epithelial cells^90–92^ and have been widely used as a model to study *S*. *pneumoniae* interaction with human cells^37,51^. A549 cells were cultured on 96-well plates (2.5 × 10^5^ cells/well) in DMEM without antibiotics. Following overnight incubation each well contained ~10^5^ A549 cells. WU2 WT, WU2Δ*flaR* ^Erm^ and WU2Δ*flaR* ^*flaR*/Erm/Kan^ strains (~10^6^ CFU; multiplicity of infection (MOI) 10:1) were added to the cells for 1 hr incubation at 37 °C. Bacteria were enumerated after the 1 h incubation and no bacterial death under the experimental conditions could be observed in WT, mutant and complemented strains (data not shown). Excess bacteria were removed, and cells detached with 0.25% trypsin-EDTA and plated onto blood agar plates for enumeration. This experiments were performed in triplicate and repeated three times.

### rFlaR elicits antibody response in immunized mice

Mice were immunized with 25 µg rFlaR refolded in the presence of 2 mM Fe^2+^ using CFA in primary immunization and IFA in the following booster immunizations. Microtiter plates (F96 Maxisorp Nunc (ThermoFisher) were coated with 1 µg/ml solution of rFlaR in bicarbonate buffer pH 9.6. Plates were blocked with 2% BSA solution in PBS. Sera obtained from mice immunized with rFlaR following each immunization were diluted with PBS and used as the primary antibodies. Washes were performed with PBS supplemented with 0.05% tween. Secondary antibody used was HRP conjugated anti-mouse IgG (Jackson laboratories). Detection was performed using tetramethylbenzidine (TMB; DakoCytomation) as substrate. Reaction was stopped using 1 M H_2_SO_4_. Absorbance was determined at 450 nm using ELISA reader multimode detector DTX 880 (BECKMAN COULTER). Serum obtained from mice immunized with rPtsA^37^ (PtsA IV) was used as a negative control.

### Statistical Analysis

The Shapiro-Wilk test was used and post-hoc statistical power was calculated, respectively, to verify that small sample sized data sets assumed normal distribution and are of the sufficient size to justify the use of one-way ANOVA for parametric data, followed by the Dunnett’s test for multiple comparisons. One-tailed Student’s *t*-test with Welch’s correction was used for bacterial load comparisons of two groups. Data was reported as the mean ± SEM, unless stated otherwise. Pearson and Spearman correlations were used to assess the significance of change. Survival of *S*. *pneumoniae*-inoculated mice was determined using Log-rank (Mantel-Cox) test. Differences were considered significant at p < 0.05. All statistical analyses were performed with the software package in GraphPad Prism version 7 (La Jolla, CA, USA).

## Electronic supplementary material

Supplementary material
