# Supplementary material for: Flavin Reductase Contributes to Pneumococcal Virulence by Protecting from Oxidative Stress and Mediating Adhesion and Elicits Protection Against Pneumococcal Challenge
Source: Sci Rep. 2018 Jan 10;8:314. doi: 10.1038/s41598-017-18645-8 (PMC5762878; doi:10.1038/s41598-017-18645-8)
Supplement: Supplementary file 1 — Supplementary material [file 41598_2017_18645_MOESM1_ESM.docx]

**Flavin Reductase Contributes to Pneumococcal Virulence by Protecting From Oxidative Stress and Mediating Adhesion and Elicits Protection Against Pneumococcal Challenge**

Giora I. Morozov^1^, Nurith Porat^1,2^, Tatyana Kushnir^1^, Hastyar Najmuldeen^3,4^, Asad Adawi^1^, Vered Chalifa-Caspi^5^, Rachel Benisty^1,2^, Ariel Ohayon^1^ Ofir Liron^6^, Shalhevet Azriel^1^, Itai Malka^1^, Shahar Dotan^6^, Maxim Portnoi^6^, Andrew A Piotrowski^7^, Daniel Kafka^6^, Barak Hajaj^1,2^, Tali Fishilevich^1^, Marilou Shagan^1^ Michael Tal^6^, Ron Ellis^6^, Donald A Morrison^7^, Andrea M Mitchell^8^, Timothy J Mitchell^8^, Ron Dagan^9^, Hasan Yesilkaya^3^, Yaffa Mizrachi Nebenzahl^1^

**Supplementary Table S1:** **Primers used in the current study.**

| Primer | Restriction site | Sequence |
| --- | --- | --- |
| *flaR* pHAT expression | BamHI  SacI | F 5′-TC**GGATCC**GTGATTGGAGTCGTTTGCG -3′  R 5′-TC**GAGCTC**TCAGTTCTTGCCATCCTTCT-3′ |
| *flaR* pET30a (+) expression | NdeI Bpu1102I | 5'-CATGGGATC**CATATG**ATTGGAGTCGTTGCGAGA-3'  5'-CATGGAGCTC**GCTCAGC**AAACGAGACGTTCTGTTTCG-3' |
| Upwing  WU2Δ*flaR* | BamHI | F 5'-CCAAGATGCCATGATTCGCTACGAGAG-3'  R 5'-AG**GGATCC**TGCTCCATCGCAGCAT-3' |
| Downwing  WU2Δ*flaR* | ApaI | F 5'-ATA**GGGCCC**GGATAAGCGTGTAGATATGAAGG-3'  R 5'-CTACAAGTTCTTTTGGCGACACTGTCAG-3' |
| Kan AB cassette | ApaI  BamHI | F 5'-GCGC**GGGCCC**CCGTTTGATTTTTAATGGATA-3'  R 5'-AATCG**GGATCC**CTAGGTACTAAAACA-3' |
| Erm AM cassette | BamHI  ApaI | F 5'-CCGC**GGATCC**AGTCGGCAGCGACT-3'  R 5'-CC**GGGCCC**AAAATTTGTTTGAT-3' |
| *flaR* pBAV-K1  and pCEP complementation | NcoI  PstI | F 5'-**CCATGG**AGTACCTATCGTGATTGGAG-3'  R 5'- **CTGCAG**TCAGTTCTTGCCATC-3' |
| *flaR* pCEP verification | Mal  pCEP | F 5'- GCTTGAAAAGGAGTATACTT -3'  R 5'- AGGAGACATTCCTTCCGTATC -3' |
| Kan resistance in pCEP verification |  | F 5'- GAGGTGCTACCATGGCGCGCA -3'  R 5'- CTAAAACAATTCATCCAGTAA -3 |

**Supplementary Fig. S1. FlaR sequence contains a three-cysteine cluster and flavin-binding domain.**
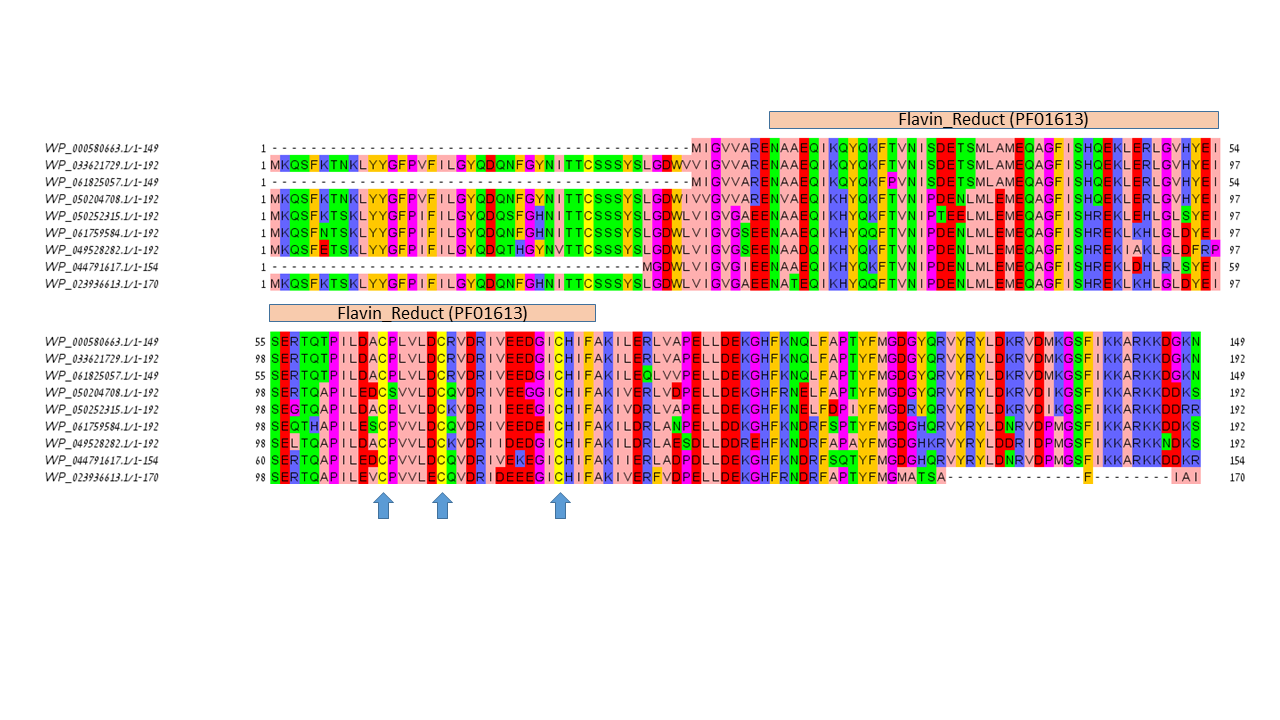


Multiple sequence alignment of a divergent set of 9 *S. pneumoniae* FlaR proteins, which were selected out of 63 FlaR proteins of this species in RefSeq. A rectangle above the alignment shows the position of PFAM Flavin Reductase domain. Arrows below the alignment show the position of conserved cysteines (equivalent to positions 66, 72 and 84 of WP_000580663.1).

**Supplementary Fig. S2. Expression of rFlaR (original scan).**


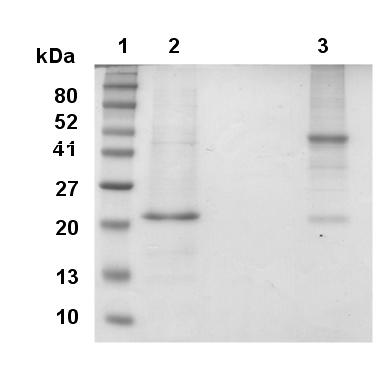


rFlaR was purified using Ni-NTA beads under denaturing conditions and resolved by SDS-PAGE using sample buffer with (lane 1) or without (lane 2) β-mercaptoethanol (β-ME). Two empty lanes were left between lane 2 and lane 3.

**Supplementary Fig. S3. Construction of WU2Δ*flaR*^Kan^ strain (original scan).**


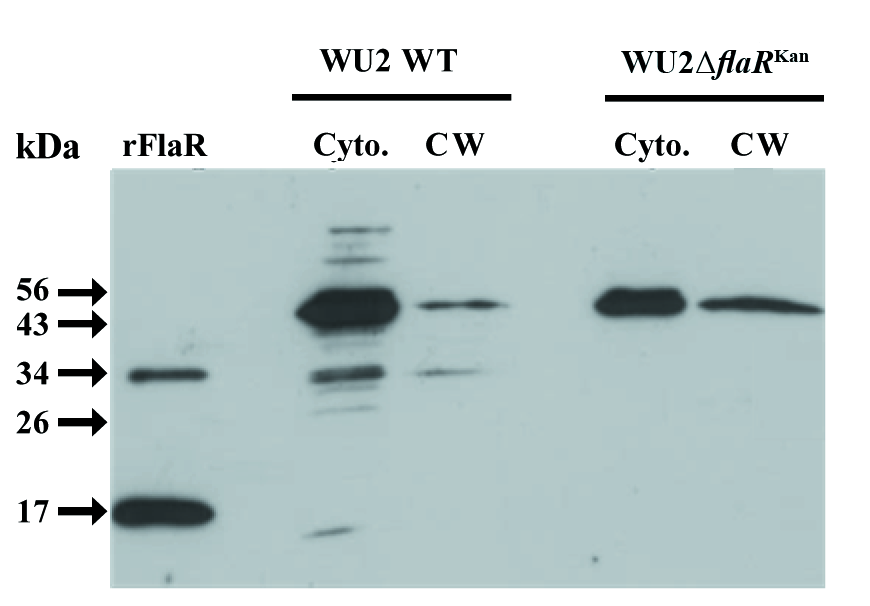


WU2Δ*flaR*^Kan^ was constructed by homologous recombination as described in the Methods section. WU2Δ*flaR*^Kan^ cell-wall (CW) and cytoplasmic (cyto) fractions were analyzed by immunoblotting and probed with rabbit anti-rFlaR antibodies.

**Supplementary Fig. S4. Growth rates of WU2, WU2Δ*flaR*^Erm^ and the complemented strains, WU2Δ*flaR^flaR^*^/Erm/Kan^ plasmid and WU2Δ*flaR^flaR^*^/Erm/Kan^ chromosome.**


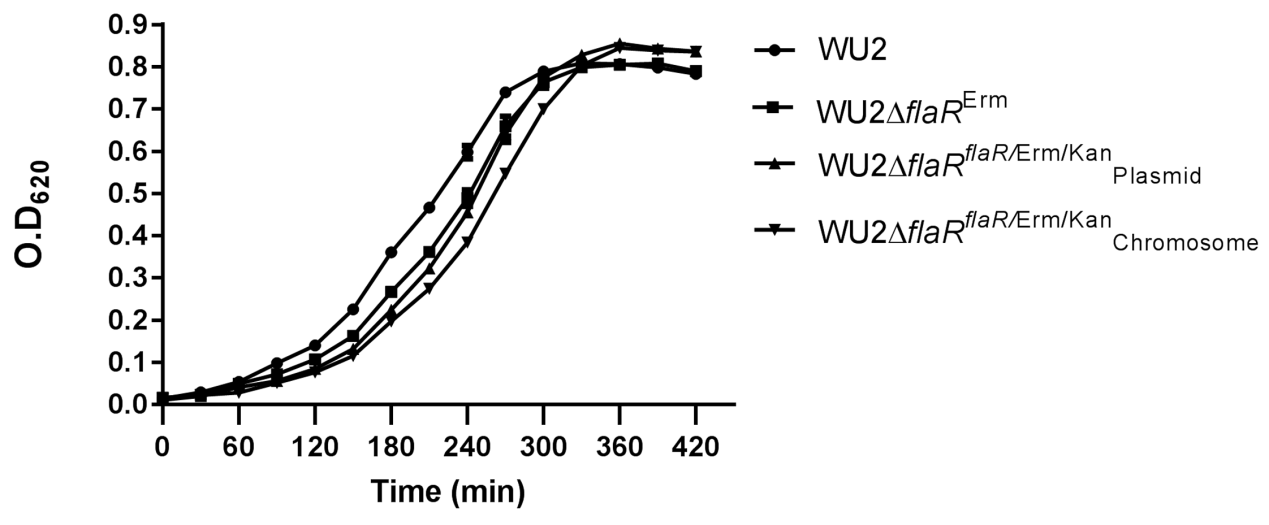


Strains were grown in 20 ml of THY under anaerobic conditions. The slopes of the data sets were calculated following linear regression fit (*S_WU2_* = 0.002358 ± 0.0001821; *S_WU2ΔflaR_^Erm^* = 0.002380 ± 0.0001748; *S_WU2ΔflaR_^flaR/Erm/Kan^ _plasmid_* = 0.002523 ± 0.0001949; *S_WU2ΔflaR_^flaR/Erm/Kan^ _chromosome_* = 0.002459 ± 0.0001907). Since the slopes are not significantly different (p =0.9192, not significant), it is possible to calculate one slope for all the data sets that equals to 0.00243.

**Supplementary Fig. S5. rFlaR elicits antibody response in immunized mice.**


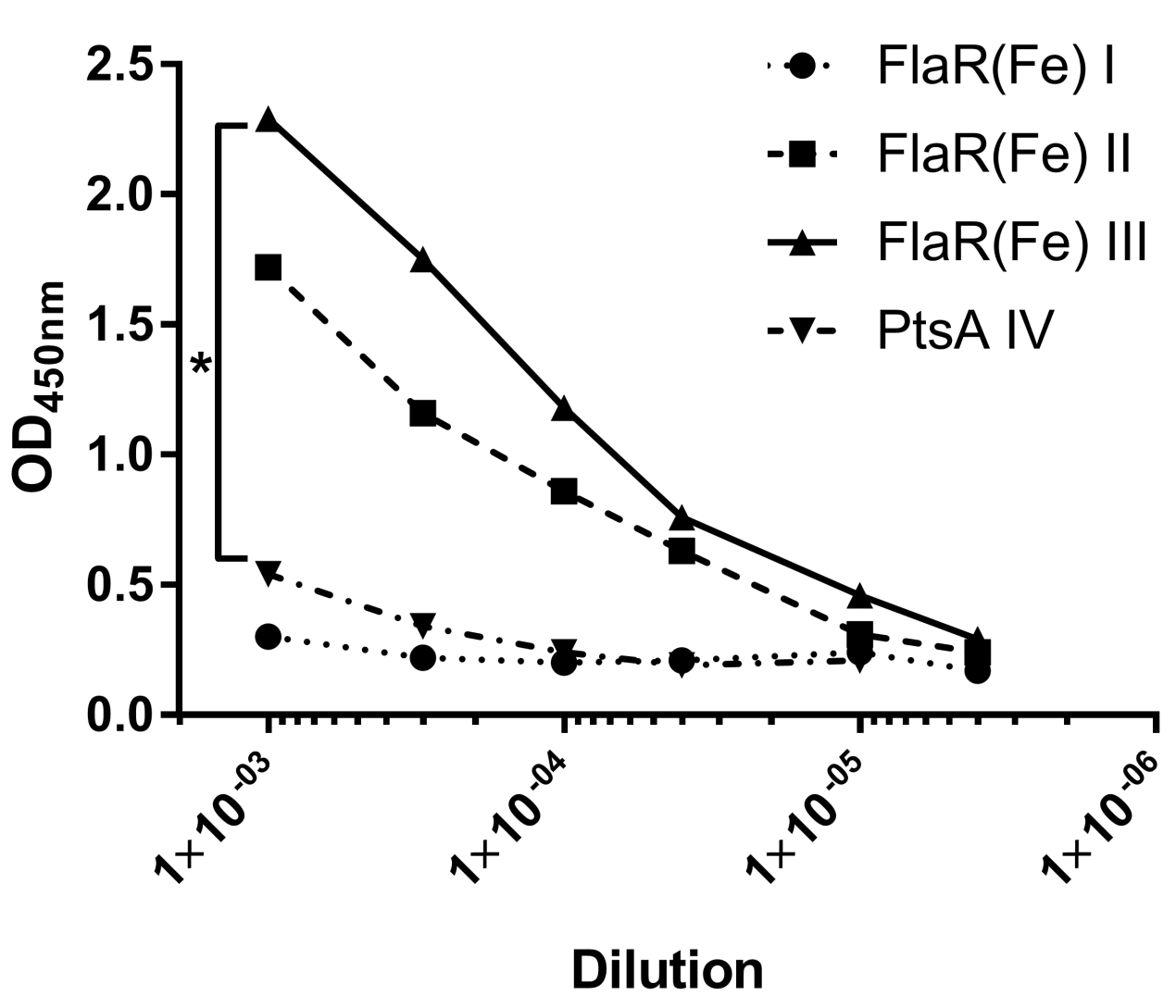


Mice were immunized with 25 µg rFlaR refolded in the presence of 2 mM Fe^2+^, using CFA in primary immunization and IFA in the second and the third booster immunization. Sera obtained after primary [FlaR (Fe) I], secondary [FlaR (Fe) II] and tertiary [FlaR (Fe) III] immunizations were analyzed by ELISA assay. Microtiter plates were coated with 1µg / ml solution of rFlaR in bicarbonate buffer pH 9.6. The sera were diluted and used as the primary antibodies. Secondary antibody used was HRP conjugated anti-mouse IgG. Detection was performed using TMB as substrate. Absorbance was determined at 450 nm. Serum obtained from mice immunized with rPtsA (PtsA IV) was used as a negative control. One way ANOVA, Sidak post-hoc test, *p = 0.014.
